# Targeting KIF18A triggers antitumor immunity and enhances efficiency of PD-1 blockade in colorectal cancer with chromosomal instability phenotype

**DOI:** 10.1038/s41420-025-02437-5

**Published:** 2025-04-02

**Authors:** Gang Liu, Yan Zhang, Zhen Cao, Zhanwei Zhao

**Affiliations:** Senior Department of General Surgery, Chinese PLA General Hospital, Beijing, China

**Keywords:** Cancer immunotherapy, Cancer microenvironment

## Abstract

Colorectal cancer with chromosomal instability (CIN^+^) phenotype is immunosuppressive and refractory to immune checkpoint blockade (ICB) therapy. Recently, KIF18A is found to be a mitotic vulnerability in chromosomally unstable cancers, but whether targeting KIF18A affects antitumor immunity in CIN^+^ colorectal cancer is unknown. In our study, western blot, cell viability assay, transwell migration and invasion assays, flow cytometry, animal model, immunohistochemistry (IHC) staining, reverse transcription–quantitative PCR (RT-qPCR) and ELISA assay were conducted to evaluate the potential function of KIF18A in CIN^+^ colorectal cancer. We found that KIF18A inhibition by short hairpin RNAs (ShRNAs) or small inhibitor AM-1882 suppressed proliferation, migration, invasion and tumor growth and metastasis of CIN^+^ colorectal cancer cells in vitro and in vivo. Moreover, targeting KIF18A disrupted cell-cycle progression and induced G2/M arrest in CIN^+^ colorectal cancer cells. In addition, KIF18A inhibition promoted immune infiltration and activation in CIN^+^ colorectal tumors. KIF18A inhibition suppressed proliferation of Tregs and increased infiltration and activation of cytotoxic CD8^+^ T cells in CIN^+^ colorectal tumors. Mechanically, KIF18A inhibition stimulated type I IFN signaling and cGAS-STING activation in CIN^+^ colorectal tumors. Finally, targeting KIF18A enhanced PD-1 blockade efficiency in CIN^+^ colorectal tumors through T cells. Our data elucidated a novel role of KIF18A in antitumor immunity of CIN^+^ colorectal cancer.

## Introduction

Colorectal cancer is the second most common cancer in women and third in men, accounting for 9.2% of cancer related death worldwide [1]. Its incidence is predicted to increase by 63% to 3.2 million at 2040 [2]. Colorectal cancer patients have a 5-year survival rate of 65%. The distribution of colorectal cancer is varied across race and ethnicity. Asia has the highest rate of colorectal cancer (52.3%), following by Europe (26.9%) and North America (9.3%) [2]. Risk factors for colorectal cancer include family history of colorectal cancer, inflammatory bowel disease, smoking, obesity, diabetes and excessive alcohol consumption [3]. Colorectal cancer is a heterogeneous disease characterized by genomic instability. There are two distinct forms of genomic instability during colorectal carcinogenesis, chromosomal instability (CIN^+^) and microstate instability (MSI). CIN is the more common form of genomic instability in colorectal cancer, occurring in 60~70% of sporadic colorectal cancer patients [4]. In comparison, MSI only takes up 15% of colorectal cancer cases. CIN^+^ colorectal cancer develops through increasing occurrence of chromosome segregation errors, loss of heterozygosity (LOH) in chromosomes, and ongoing numerical and structural chromosomal aberrations [5]. MSI^+^ colorectal cancer is developed by the deficiency in mismatch repair apparatus. Notably, CIN^+^ colorectal cancer patients have a worse prognosis than colorectal patients with MSI.

Emerging evidences indicate that CIN high tumors have an immunosuppressive phenotype [6]. According to an extensive immunogenomic analysis of more than 10,000 tumors comprising 33 diverse cancer types from TCGA, aneuploidy is positively associated with increasing immune-suppressive macrophage infiltration and TGF-β pathway activation [7]. Moreover, genomic copy-number alternations are correlated with changes in tumor immune composition. For example, 1p amplification is positively correlated with increasing leukocyte infiltration, while loss of 19q that harboring TGFB1 results in reducing leukocyte infiltration [7]. In addition, highly aneuploid tumors exhibit less expression of markers for cytotoxic cells and natural killer cells, and more infiltration of M2 tumor-associated macrophages, resulting in reduced antitumor immunity [8]. In colorectal cancer, high-CIN tumors show an immune exclusion feature that predict poor immune checkpoint blockade (ICB) response [9]. Indeed, high-CIN colorectal tumors are “immune cold”, less responsive to anti-PD-1 therapy, and correlates with poor survival, while MSI colorectal tumors are more responsive to anti-PD-1 therapy and predict a better outcome [9, 10]. Therefore, there is an urging needing to explore the molecular vulnerability for high-CIN colorectal tumors and develop novel strategy to enhance ICB response.

KIF18A (Kinesin family member 18A) belongs to the kinesin superfamily of microtubule-associated molecular motors and plays a critical role in cell division, mitotic metaphase and anaphase. Accumulated studies demonstrate that KIF18A is a genetic vulnerability of high-CIN tumors, and targeting KIF18A suppresses growth and metastasis of CIN^+^ tumors [11]. For instance, using the sequencing data from nearly 10,000 primary human cancer samples and more than 600 cancer cell lines, KIF18A is found to be a genetic vulnerability for whole-genome doubling cancer cells and silencing KIF18A induces mitotic errors and growth arrest [12]. Moreover, aneuploid cancer cells are particularly vulnerable to KIF18A inhibition, while KIF18A overexpression increases the sensitivity to spindle assembly checkpoint inhibition [13]. CIN^+^ tumor cells specifically require KIF18A for proliferation, and KIF18A inhibition in CIN^+^ tumor cells show mitotic delays, multipolar spindles, and cell apoptosis [14]. In addition, small inhibitors targeting KIF18A are particularly effective in tumor cells with CIN feature [11].

Accumulated studies reveal that targeting cell-cycle proteins may play additional roles in tumor development by affecting tumor microenvironment and antitumor immune response [15]. For example, CDK4/6 inhibition not only induces cell-cycle arrest, but also promotes antitumor immunity by stimulating IFN signaling, suppressing regulatory T (Treg) cell proliferation, and enhancing cytotoxic T cell-mediated clearance of tumor cells [16]. Although previous studies reveal that KIF18A is a marker for prognosis and immunity of various cancers [17, 18], it is uncertain if KIF18A inhibition may affect antitumor immunity of CIN^+^ colorectal tumors. In the present study, the potential influence of KIF18A on immune infiltration in CIN^+^ colorectal tumor was evaluated. We found that KIF18A inhibition by a small inhibitor AM-1882 promoted immune infiltration and activation in CIN^+^ colorectal tumors. Moreover, KIF18A inhibition stimulated type I IFN signaling and cGAS-STING activation. Finally, targeting KIF18A enhanced response to anti-PD-1 immunotherapy through T cells. Our data provided a novel role of KIF18A in antitumor immunity of CIN^+^ colorectal cancer.

## Results

### KIF18A inhibition suppresses growth and metastasis of CIN^+^ colorectal cancer cells

**Fig. 1:**
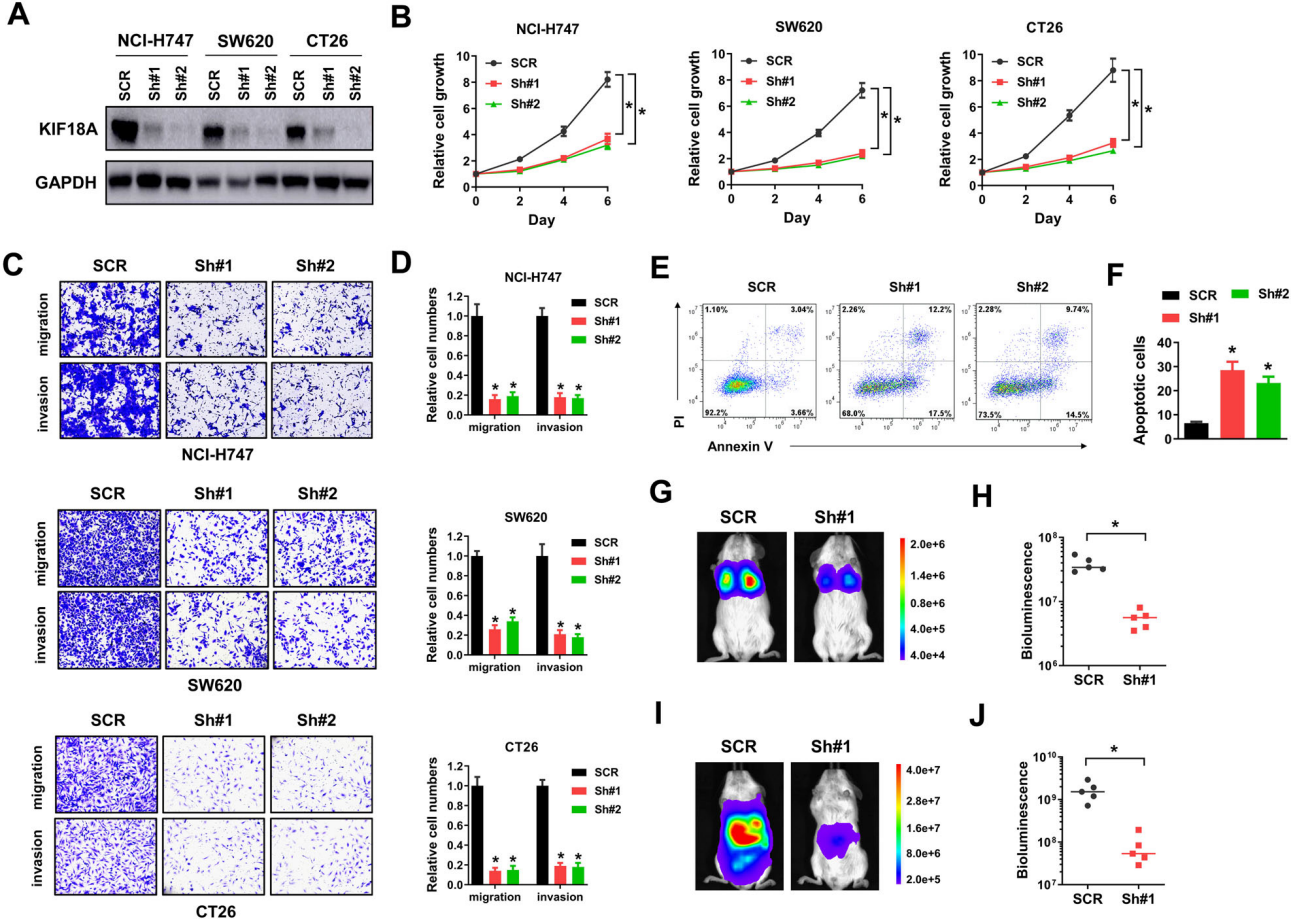
Silencing KIF18A inhibits growth, migration, invasion and metastasis of CIN^+^ colorectal cancer cells. **A** NCI-H747, SW620 and CT26 cells were introduced with SCR, Sh#1 and Sh#2 lentivirus, then collected lysates for western blot. **B** NCI-H747, SW620 and CT26 cells introducing with SCR, Sh#1 and Sh#2 lentivirus were seeded into 96-well-plates (3000/well), then cell viability was determined by Cell Counting Kit-8 at day 2, 4 and 6. **C**, **D** NCI-H747, SW620 and CT26 cells introducing with SCR, Sh#1 and Sh#2 lentivirus were used for transwell migration and invasion assays. Representative images (**C**) and relative migration and invasion cells (**D**) were shown. E-F, CT26 cells introducing with SCR, Sh#1 and Sh#2 lentivirus were stained with PI and Annexin V-FITC for flow cytometry. Representative plots (**E**) and percentage of apoptotic cells (**F**) were shown. G-J, CT26 cells introducing with SCR or Sh#1 lentivirus were injected into the tail vein and spleen of ICR mice, then lung and liver metastasis were evaluated. Representative bioluminescence images (**G**, **I**) and bioluminescence signals (**H**, **J**) were shown. *P < 0.05.

**Fig. 2:**
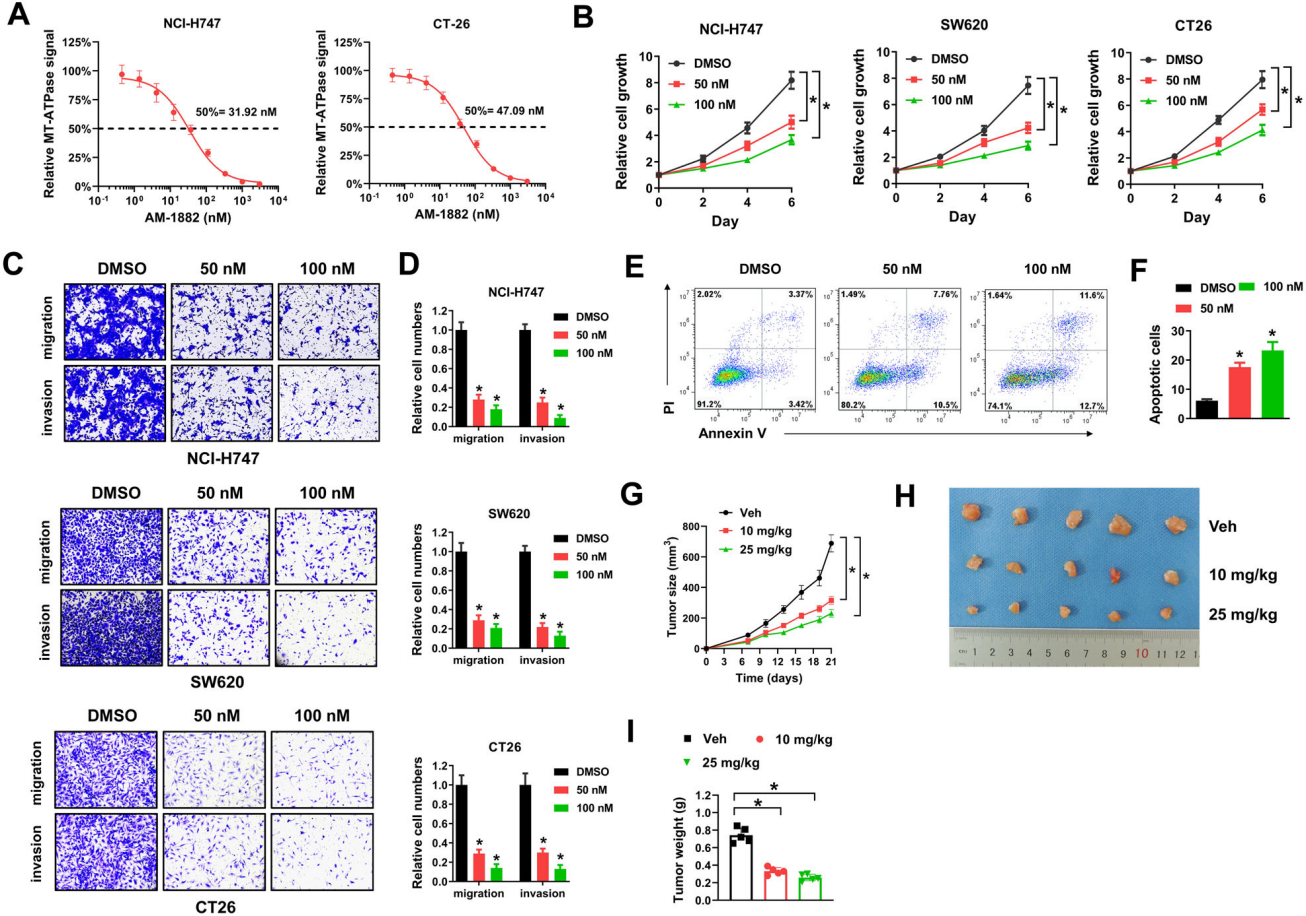
KIF18A inhibitor AM-1882 suppresses growth, migration, invasion and tumor xenograft formation of CIN^+^ colorectal cancer cells. **A** NCI-H747 and CT26 cells were treated with AM-1882 for KIF18A motor assay. Relative MT-ATPase luminescence signal compared with DMSO control was shown. **B** NCI-H747, SW620 and CT26 cells were treated with 50 nM or 100 nM AM-1882 or equal volume of DMSO, then seeded into 96-well plates (3000/well) for cell viability assay. **C**, **D** NCI-H747, SW620 and CT26 cells treating with 50 nM or 100 nM AM-1882 or equal volume of DMSO were used for transwell migration and invasion assays. Representative images (**C**) and relative migration and invasion cells (**D**) were shown. CT26 cells treating with 50 nM or 100 nM AM-1882 or equal volume of DMSO were stained with PI and Annexin V-FITC for flow cytometry. Representative plots (**E**) and percentage of apoptotic cells (**F**) were shown. G-I, CT26 cells (5.0 × 10^5^) were subcutaneously injected into 6-week-old female ICR mice (n = 5 for each group), then treated with 10 mg/kg or 25 mg/kg AM-1882 by gavage every other day for three weeks. Tumor growth curves (**G**), representative tumor images (**H**) and tumor weight (**I**) were shown. *P < 0.05.

To evaluate the potential influence of KIF18A inhibition, shRNAs specifically targeting KIF18A (Sh#1 and Sh#2) were constructed and introduced into chromosome instability (CIN^+^) colorectal cancer cells. Human colorectal cancer cell lines NCI-H747 and SW620, and mouse colorectal cancer cell line CT26 are proved to be CIN^+^ while human cancer colorectal cancer cell line HCT-116 and mouse colorectal cancer cell line MC38 are diploid or nearly diploid (CIN^−^) [19–21]. In our study, introducing with these two shRNAs successfully depleted KIF18A expression in NCI-H747, SW620 and CT26 CIN^+^ colorectal cancer cells compared with scramble control (SCR) (Fig. 1A). Moreover, KIF18A knockdown by these two shRNAs evidently suppressed cell growth, migration and invasion of NCI-H747, SW620 and CT26 CIN^+^ colorectal cancer cells (Fig. 1B–D). In contrast, KIF18A knockdown enhanced cell apoptosis in CT26 cells (Fig. 1E, F). Furthermore, KIF18A knockdown apparently reduced lung and liver metastasis of CT26 cells in immune-competent ICR mice (Fig. 1G–J). Next, AM-1882, a small inhibitor selectively targeting KIF18A, was used in our study. AM-1882 is demonstrated to inhibit the MT-ATPase motor activity of KIF18A and has minimal detrimental effects on healthy cells [11]. Firstly, we verified the influence of AM-1882 on the MT-ATPase motor activity of KIF18A. AM-1882 evidently reduced the MT-ATPase motor activity of KIF18A dose-dependently in NCI-H747 and CT26 cells (Fig. 2A). Next, AM-1882 was proved to suppress cell growth, migration and invasion of NCI-H747, SW620 and CT26 CIN^+^ colorectal cancer cells compared with cells treated with vehicle control (DMSO) (Fig. 2B–D). AM-1882 treatment caused cell apoptosis in CT26 cells (Fig. 2E, F). In ICR mice, AM-1882 treatment evidently suppressed tumor formation of CT26 cells (Fig. 2G–I). The potential influence of KIF18A inhibition was also tested in HCT-116 and MC38 CIN^-^ colorectal cancer cells. However, silencing KIF18A showed no apparent influence on growth, migration, invasion and apoptosis of HCT-116 and MC38 cells (Supplementary Fig. 2A–E). Taken together, our results indicated that KIF18A inhibition suppressed growth and metastasis of CIN^+^ colorectal cancer cells.

### Targeting KIF18A disrupts cell-cycle progression of CIN^+^ colorectal cancer cells

**Fig. 3:**
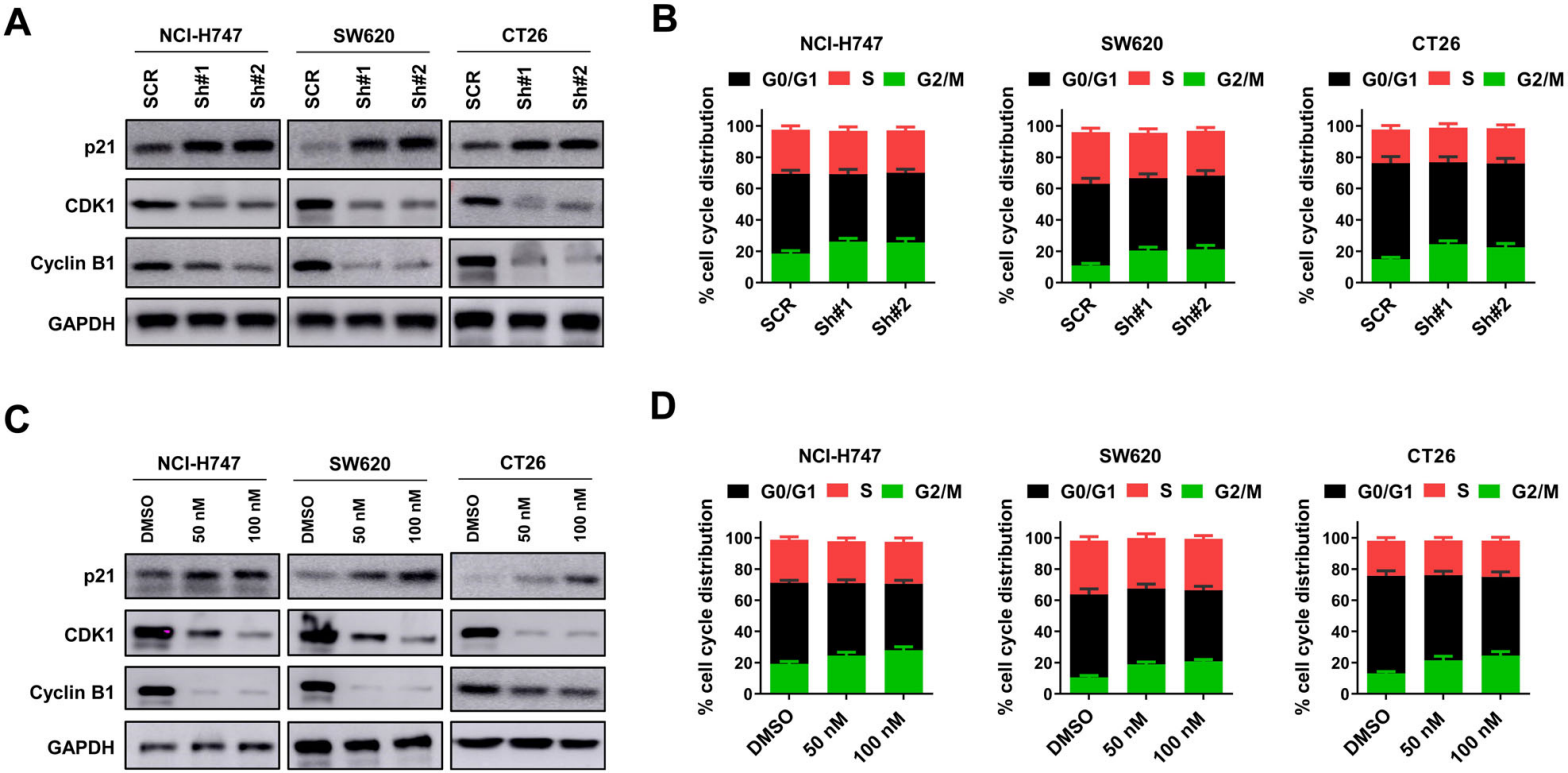
Targeting KIF18A disrupts cell-cycle progression of CIN^+^ colorectal cancer cells. **A** NCI-H747, SW620 and CT26 cells were introduced with SCR, Sh#1 and Sh#2 lentivirus, then collected lysates for western blot. **B** NCI-H747, SW620 and CT26 cells introducing with SCR, Sh#1 and Sh#2 lentivirus were stained with PI for flow cytometry. The percentages of cells in G0/G1, S and G2/M phases were shown. **C** NCI-H747, SW620 and CT26 cells were treated with 50 nM or 100 nM AM-1882 or equal volume of DMSO, then collected lysates for western blot. **D** NCI-H747, SW620 and CT26 cells treating with 50 nM or 100 nM AM-1882 or equal volume of DMSO were stained with PI for flow cytometry. The percentages of cells in G0/G1, S and G2/M phases were shown. *P < 0.05.

The possible influence of KIF18A inhibition on cell-cycle progression of CIN^+^ colorectal cancer cells was further evaluated. KIF18A knockdown increased the protein expression of p21, and reduced the protein expression of CDK1 and cyclin B1 in NCI-H747, SW620 and CT26 CIN^+^ colorectal cancer cells, suggesting that KIF18A inhibition triggered G2/M phase arrest (Fig. 3A). This was further validated by flow cytometry. KIF18A knockdown increased the percentage of G2/M cells rather than G0/G1 and S phases in NCI-H747, SW620 and CT26 CIN^+^ colorectal cancer cells (Fig. 3B). Similarly, AM-1882 treatment promoted the protein expression of p21, and suppressed the protein expression of CDK1 and cyclin B1 in NCI-H747, SW620 and CT26 CIN^+^ colorectal cancer cells (Fig. 3C). Meanwhile, AM-1882 treatment caused more cells arresting in G2/M phase (Fig. 3D). In contrast, silencing KIF18A showed no apparently influence on protein expression of p21, CDK1 and cyclin B1 in HCT-116 and MC38 CIN^-^ colorectal cancer cells (Supplementary Fig. S2A). Meanwhile, silencing KIF18A showed no apparent influence on the percentage of G2/M cells in HCT-116 and MC38 (Supplementary Fig. 2B). Above all, these data indicated that targeting KIF18A disrupted cell-cycle progression and induced G2/M arrest in CIN^+^ colorectal cancer cells.

### KIF18A inhibition enhances immune infiltration and activation in CIN^+^ colorectal tumors

**Fig. 4:**
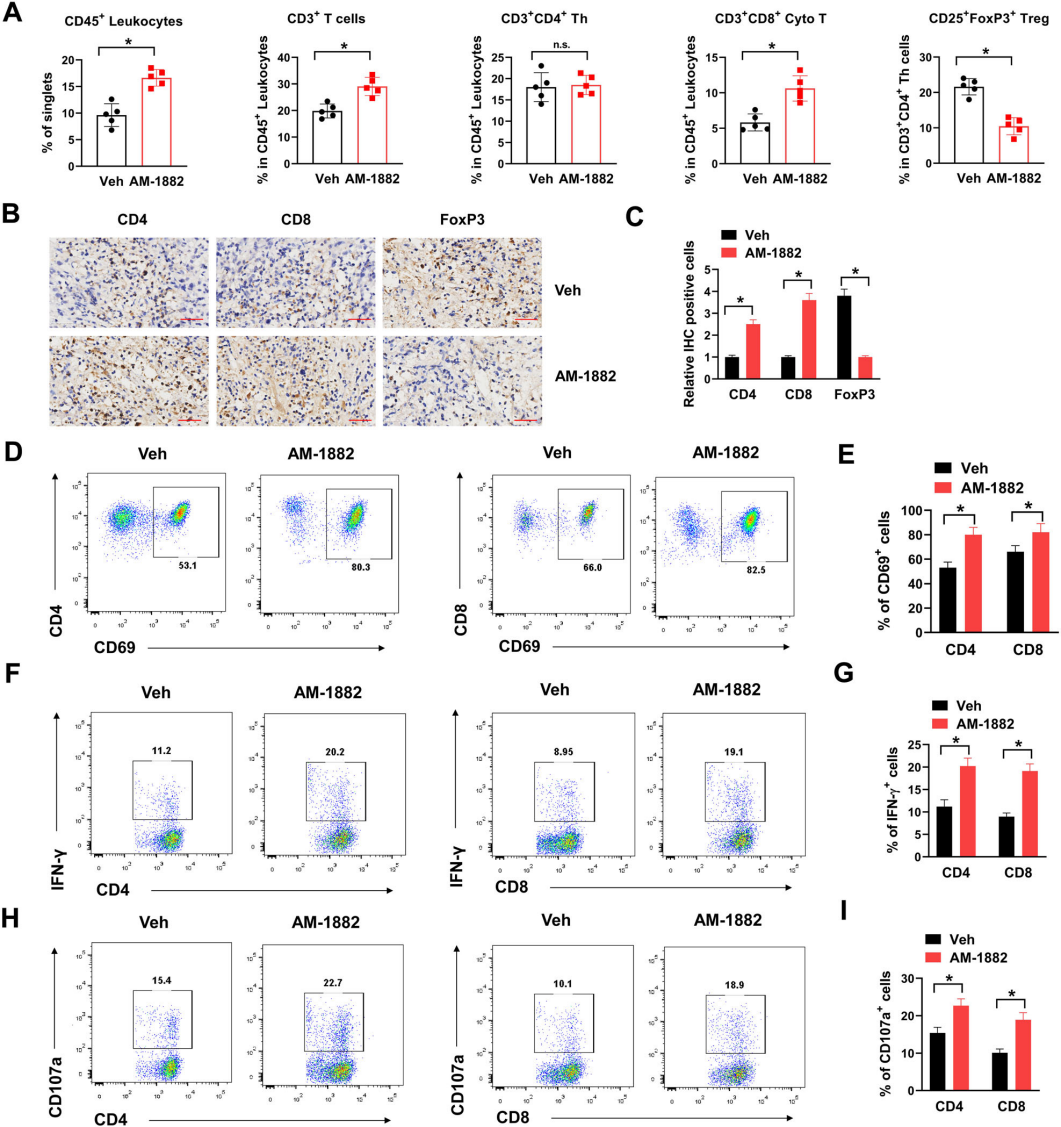
KIF18A inhibition promotes immune infiltration and activation in CIN^+^ colorectal tumors. **A** The percentages of infiltrating CD45^+^ leukocytes, CD3^+^ T cells, CD3^+^CD4^+^ Th cells, CD3^+^CD8^+^ Cyto T cells, and CD25^+^FoxP3^+^ Treg cells in CT26 tumors treating with 25 mg/kg AM-1882 or vehicle control (Veh) were shown. **B**, **C** IHC staining of CD4, CD8 and FoxP3 in CT26 tumors treating with 25 mg/kg AM-1882 or vehicle control (Veh). Representative images (**B**) and relative IHC-positive cells (**C**) were shown. The percentages of CD69 (**D**, **E**), IFN-γ (**F**, **G**) and CD107a (**H**, **I**) positive cells in infiltrating CD4^+^ and CD8^+^ T cell subsets of CT26 tumors were shown. Representative plots (**D**, **F**, **H**) and percentages of CD69, IFN-γ and CD107a positive cells (**E**, **G**, **I**) were shown. *P < 0.05.

Emerging evidences indicate that targeting cell-cycle machinery in cancer may not only influence tumor cells, but also their microenvironment, including antitumor immune response [15]. To evaluate the influence of KIF18A inhibition on immune infiltration in CIN^+^ colorectal tumors, CT26 cells were subcutaneously injected into immune-competent ICR mice and treated with AM-1882 for three weeks. At the end of drug treatment, tumors were dissected out, then infiltrating immune cells were evaluated by flow cytometry. Representative gating strategy for flow cytometry analysis was depicted in Supplementary Fig. 3. We found that AM-1882 treatment significantly increased the percentage of infiltrating CD45^+^ leukocytes, CD3^+^ T cells, CD3^+^CD8^+^ Cytotoxic T (Cyto T) cells, and decreased the percentage of CD25^+^FoxP3^+^ regulator T (Treg) cells in CT26 tumors compared with vehicle control (Veh), suggesting that KIF18A inhibition enhanced immune infiltration (Fig. 4A). This was further validated by IHC staining. There were more CD4 and CD8 positive cells and less FoxP3 positive cells after AM-1882 treatment (Fig. 4B, C). Furthermore, knockdown of KIF18A also increased the percentage of infiltrating CD45^+^ leukocytes, CD3^+^ T cells, CD3^+^CD8^+^ Cytotoxic T (Cyto T) cells, and decreased the percentage of CD25^+^FoxP3^+^ regulator T (Treg) cells in CT26 tumors compared with scramble control (Scr) (Supplementary Fig. 4A). CD69 is a T cell activation marker. AM-1882 treatment significantly increased the percentage of CD69^+^ cells in infiltrating CD4^+^ and CD8^+^ T cell subsets in CT26 tumors (Fig. 4D, E). IFN-γ and CD107a are effector T cell markers. The positive rate of IFN-γ and CD107a in infiltrating CD4^+^ and CD8^+^ T cell subsets was dramatically upregulated in AM-1882 treated CT26 tumors (Fig. 4F–I). Notably, AM-1882 treatment showed no apparent influence on the percentage of CD3^+^ T cells, CD3^+^CD4^+^ Th cells, CD3^+^CD8^+^ Cyto T cells, CD69^+^CD4^+^ T cells and CD69^+^CD8^+^ T cells in spleen, liver, lung and colon tissues of mice bearing with/without CT26 tumors (Supplementary Fig. 4B, C). To confirm that the effect of AM-1882 on infiltrating immune cells was limited in CIN^+^ colorectal tumors, CIN^−^ colorectal MC38 cells were subcutaneously injected into immune-competent ICR mice, then treated with AM-1882 for three weeks. However, AM-1882 treatment showed no evident influence on the infiltrating of CD45^+^ leukocytes, CD3^+^ T cells, CD3^+^CD4^+^ Th cells, CD3^+^CD8^+^ Cyto T cells and CD25^+^FoxP3^+^ Treg cells in MC38 tumors (Supplementary Fig. 5A). Furthermore, AM-1882 treatment did not change the percentage of CD69^+^ and IFN-γ^+^ cells in infiltrating CD4^+^ and CD8^+^ T cell subsets of MC38 tumors (Supplementary Figure 5B, C). In summary, these results suggested that KIF18A inhibition promoted immune infiltration and activation in CIN^+^ colorectal tumors.

### KIF18A inhibition augments antitumor immunity in CIN^+^ colorectal tumors

**Fig. 5:**
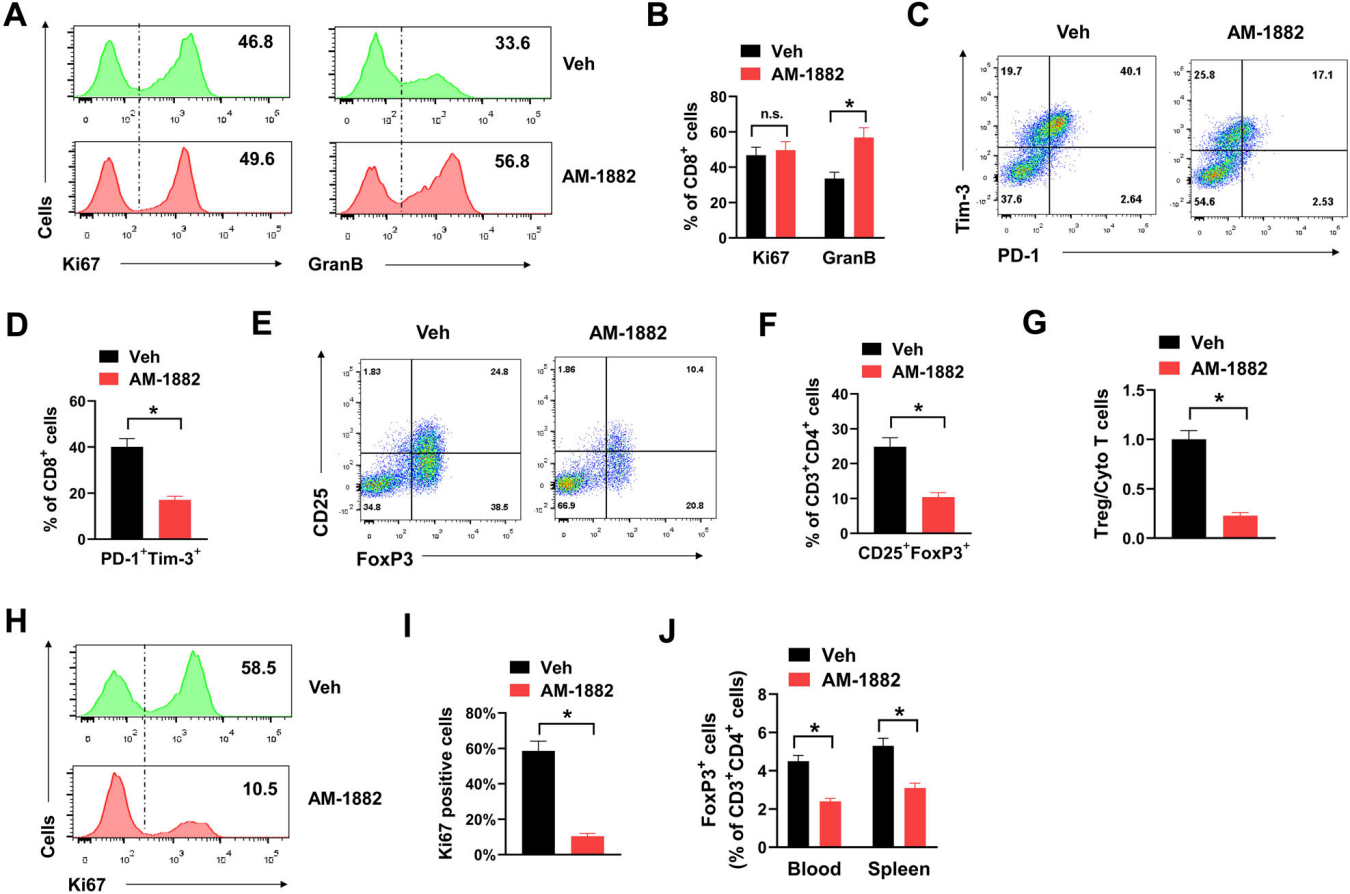
AM-1882 treatment promotes cytotoxic T infiltration and suppresses Treg infiltration in CIN^+^ colorectal tumors. **A** infiltrating CD8^+^ T cell subsets in CT26 tumors treating with 25 mg/kg AM-1882 or vehicle control (Veh) were stained with Ki67 or Granzyme B (GranB) for flow cytometry. Representative histogram (**A**) and percentages of positive cells (**B**) were shown. **C**, **D**, infiltrating CD8^+^ T cell subsets in CT26 tumors treating with 25 mg/kg AM-1882 or vehicle control (Veh) were stained with PD-1 and TIM-3 for flow cytometry. Representative plots (**C**) and percentages of PD-1^+^ TIM3^+^ cells (**D**) were shown. **E**, **F** Infiltrating CD3^+^CD4^+^ T cell subsets in CT26 tumors treating with 25 mg/kg AM-1882 or vehicle control (Veh) were stained with CD25 and FoxP3 for flow cytometry. Representative plots (**E**) and percentages of CD25^+^ FoxP3^+^ cells (**F**) were shown. G, the ratio of Treg/Cyto T cells in CT26 tumors treating with 25 mg/kg AM-1882 or vehicle control (Veh) was shown. **H**, **I** Infiltrating Treg cell subsets in CT26 tumors treating with 25 mg/kg AM-1882 or vehicle control (Veh) were stained with Ki67 for flow cytometry. Representative histogram (H) and percentages of positive cells (**I**) were shown. **J** The percentages of Treg cells in blood and spleen of CT26 tumor-bearing mice were shown. *P < 0.05.

The influence of KIF18A inhibition on antitumor immunity of CT26 tumors was further evaluated. Ki67 and granzyme B are markers for the proliferation and cytotoxicity of CD8^+^ T cells. We found that AM-1882 treatment showed no apparent influence on the expression of Ki67, but evidently increased the expression of granzyme B in infiltrating CD8^+^ T cells of CT26 tumors (Fig. 5A, B). However, AM-1882 treatment showed no influence on the expression of both Ki67 and granzyme B expression in CD8^+^ T cells in vitro, suggesting that AM-1882 had no direct influence on T cell proliferation and activation (Supplementary Fig. 6A, B). In addition, AM-1882 also showed no influence on the percentage of CD86^+^ and MHCII^+^ cells in CD11c^+^ dendritic cells, indicating that AM-1882 did not change the maturation of dendritic cells in vitro (Supplementary Fig. 6C, D). PD-1 and Tim-3 are markers for exhausted CD8^+^ T cells. AM-1882 treatment apparently reduced the percentage of PD-1^+^Tim-3^+^ exhausted CD8^+^ T cells in CT26 tumors (Fig. 5C, D). These data indicated that KIF18A inhibition enhanced the antitumor immunity of infiltrating CD8^+^ T cells. Tregs are the dominant subpopulation of immunosuppressive T cells. In our study, AM-1882 treatment apparently reduced the percentage of CD25^+^FoxP3^+^ Treg cells in CT26 tumors (Fig. 5E, F). Thus, the ratio of Treg/Cyto T cells was significantly decreased by AM-1882 treatment, indicating a tipping of immune balance towards antitumor immunity (Fig. 5G). Furthermore, AM-1882 treatment apparently reduced the percentage of Ki67^+^ Tregs in CT26 tumors, suggesting that AM-1882 restrained the proliferation of infiltrating Tregs (Fig. 5H, I). Moreover, AM-1882 also reduced the percentage of Tregs in blood and spleen of CT26 tumor-bearing mice (Fig. 5J). To confirm the influence of KIF18A inhibition in antitumor immunity of CIN^+^ colorectal tumors, we also used another KIF18A inhibitor VLS-1488. Similarly, VLS-1488 treatment exhibited no influence on the expression of Ki67, but promoted the expression of granzyme B in infiltrating CD8^+^ T cells of CT26 tumors (Supplementary Fig. 6E, F). Meanwhile, VLS-1488 treatment also reduced the percentage of PD-1^+^Tim-3^+^ exhausted CD8^+^ T cells and CD25^+^FoxP3^+^ Treg cells in CT26 tumors (Supplementary Fig. 6G–J). Taken together, the above results suggested that KIF18A inhibition augmented antitumor immunity in CIN^+^ colorectal tumors.

### KIF18A inhibition stimulates type I IFN signaling and cGAS-STING activation in CIN^+^ colorectal tumors

**Fig. 6:**
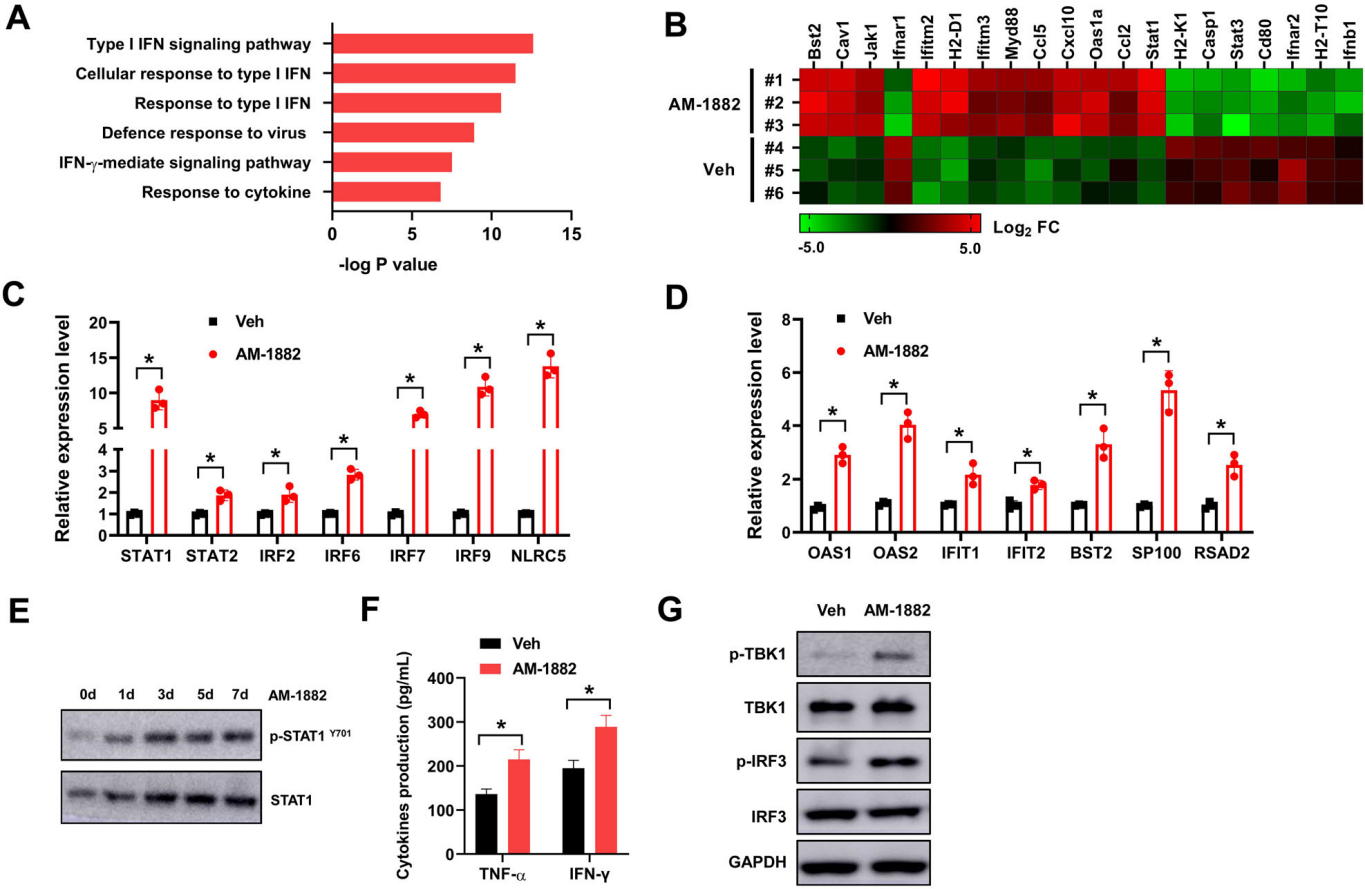
KIF18A inhibition stimulates IFN-γ signaling and activates cGAS-STING pathway in CIN^+^ colorectal tumors. **A** Genome-wide transcriptomic analysis of CT26 tumors was conducted. The top upregulated functional pathways were analyzed by GO analysis. **B** heatmap showed the top differentially expressed genes in CT26 tumors treating with 25 mg/kg AM-1882 or vehicle control (Veh). Relative expression of IFN-related transcription factors (**C**) and IFN-stimulated genes (**D**) was evaluated by RT-qPCR. E, the phosphorylated and total STAT1 protein levels were elevated in AM-1882 treated CT26 cells. **F** The production of TNF-α and IFN-γ in CT26 tumors treating with 25 mg/kg AM-1882 or vehicle control (Veh) was evaluated by ELISA assay. **G** The levels of phosphorylation and total TBK1 and IRF3 in CT26 tumors treating with 25 mg/kg AM-1882 or vehicle control (Veh) was evaluated by western blot. *P < 0.05.

To elucidate the molecular mechanism of KIF18A inhibition in antitumor immunity of CIN^+^ colorectal tumors, genome-wide transcriptomic analysis of CT26 tumors was conducted. Analysis of gene ontology (GO) categories revealed that AM-1882 treatment upregulated expression of genes enriched in GO terms related to type I IFN signaling (Fig. 6A). Some of the top differentially expressed genes were depicted in the heatmap (Fig. 6B). AM-1882 treatment induced expression of genes involved in antiviral response (Bst2, Ifnb1, Oas1a), type I IFN response (Ifnar1, Ifnar2, Stat1, Jak2 and Myd88) and chemoattractant molecules (Ccl5, Cxcl10 and Ccl2) (Fig. 6B). Moreover, expression of IFN-related transcription factors (STAT1, STAT2, IRF2, IRF6, IRF7, IRF9 and NLRC5) were apparently increased by AM-1882 treatment or KIF18A knockdown in CT26 tumors (Fig. 6C, Supplementary Fig. 7A). AM-1882 treatment and KIF18A knockdown also upregulated the expression of IFN-stimulated genes (OAS1, OAS2, IFIT1, IFIT2, BST2, SP100 and RSAD2) in CT26 tumors (Fig. 6D, Supplementary Fig. 7B). In western blot analysis, the phosphorylated and total STAT1 protein levels were elevated in AM-1882 treated CT26 cells (Fig. 6E). The levels of pro-inflammatory cytokines TNF-α and IFN-γ were evaluated by ELISA assay. AM-1882 treatment and KIF18A knockdown enhanced the production of TNF-α and IFN-γ in CT26 tumors (Fig. 6F, Supplementary Fig. 7C). The cyclic GMP-AMP synthase (cGAS)-stimulator of interferon genes (STING) pathway is a critical sensor for cytosolic double-strand DNA (dsDNA) to promote antitumor response [22]. In our study, both AM-1882 treatment and KIF18A knockdown increased the phosphorylation of TBK1 and IRF3 in CT26 tumors, suggesting that AM-1882 promoted cGAS-STING pathway activation (Fig. 6G, Supplementary Fig. 7D). Above all, our data indicated that KIF18A inhibition stimulated type I IFN signaling and cGAS-STING activation in CIN^+^ colorectal tumors.

### Targeting KIF18A enhances response to anti-PD-1 immunotherapy through T cells in CIN^+^ colorectal tumors

**Fig. 7:**
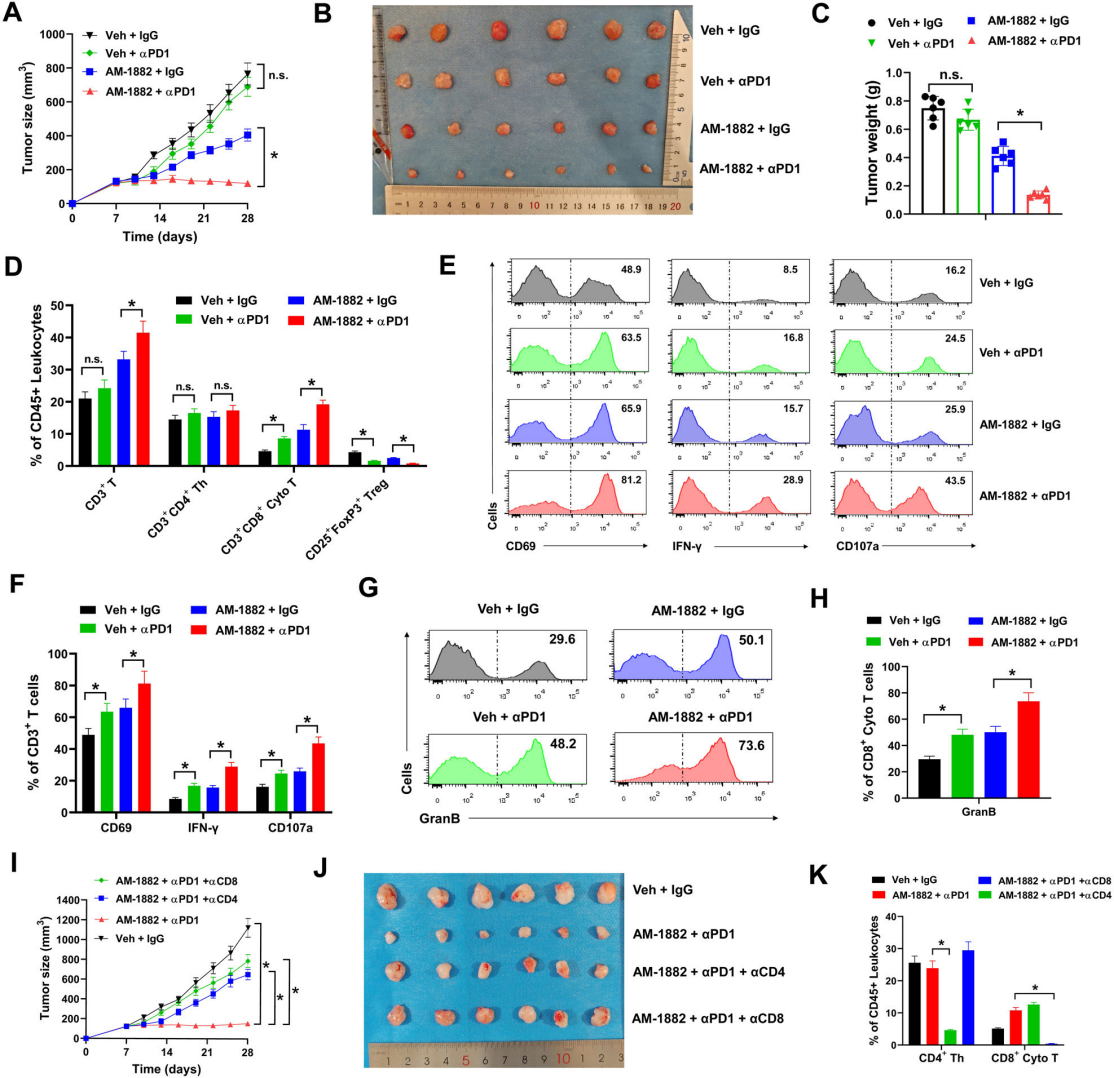
AM-1882 increases anti-PD-1 efficiency through T cells in CIN^+^ colorectal tumors. **A**–**H** ICR mice (*n* = 6 for each group) were implanted with CT26 cells for one week, then treated with the combinations of Vehicle (Veh), 10 mg/kg AM-1882, 250 μg IgG control or 250 μg anti-PD-1 mAb (αPD-1) as indicated every 3 days for 7 times as indicated. Tumor growth curves (**A**), representative images (**B**) and tumor weight (**C**) were shown. Infiltrating immune cells were evaluated by flow cytometry (**D**). The percentages of CD69, IFN-γ and CD107a positive cells in infiltrating CD3^+^ T cell subsets (**E**, **F**) and the percentage of granzyme B^+^ subset in CD8^+^ Cyto T cells (**G**, **H**) of CT26 tumors were evaluated by flow cytometry and shown. **I**–**K** ICR mice (*n* = 6 for each group) were implanted with CT26 cells for one week, then treated with the combinations of Vehicle (Veh), 10 mg/kg AM-1882, 250 μg IgG control, 250 μg anti-PD-1 mAb (αPD1), 250 μg anti-CD4 (αCD4) and 250 μg anti-CD8 (αCD8) as indicated every 3 days for 7 times. Tumor growth curves (**I**), representative images (**J**) and tumor weight (**K**) were shown. *P < 0.05.

The above results indicated that KIF18A inhibition enhanced antitumor immune response in CIN^+^ colorectal tumors. This promoted us to investigate whether KIF18A inhibition could augment anti-PD-1 immunotherapy in vivo. In our study, ICR mice were implanted with CT26 cells for one week, then treated with the combinations of Vehicle (Veh), AM-1882, IgG control or anti-PD-1 mAb (αPD-1) as indicated for three weeks. We found that anti-PD-1 mAb treatment alone showed no evident influence on growth of CT26 tumors, while AM-1822 treatment showed moderate inhibition on growth of CT26 tumor (Fig. 7A–C). However, the combination of anti-PD-1 mAb and AM-1882 evidently suppressed the growth of CT26 tumors, indicating that KIF18A inhibition enhanced response to anti-PD-1 immunotherapy (Fig. 7A–C). Flow cytometry analysis indicated that the combination of anti-PD-1 mAb and AM-1882 obviously increased the infiltration of CD3^+^ T cells and CD3^+^CD8^+^ Cyto T cells, and decreased the infiltration of CD25^+^FoxP3^+^ Tregs in CT26 tumors compared with the combination of AM-1882 and IgG control (Fig. 7D). Moreover, the combination of anti-PD-1 mAb and AM-1882 significantly increased the percentage of CD69^+^, IFN-γ^+^ and CD107a^+^ subsets in infiltrating CD3^+^ T cells of CT26 tumors, indicating enhanced T cell activation (Fig. 7E, F). Furthermore, the combination of anti-PD-1 mAb and AM-1882 upregulated the percentage of granzyme B^+^ subset in CD8^+^ Cyto T cells, indicating enhanced antitumor cytotoxicity (Fig. 7G, H). To confirm that the predominant antitumor response of the combine treatment of anti-PD-1 mAb and AM-1882 was depended on T cells, we depleted either CD4^+^ T cells or CD8^+^ T cells using anti-CD4 mAb (αCD4) and anti-CD8 mAb (αCD8) in ICR mice (Fig. 7I). Our data indicated that depletion of either CD4^+^ T cells or CD8^+^ T cells completely ablated the antitumor activity of the combine treatment of anti-PD-1 mAb and AM-1882 in CT26 tumors, suggesting that AM-1882 enhanced the effect of PD-1 blockade through T cells (Fig. 7J, K). Altogether, these results indicated that targeting KIF18A enhanced response to anti-PD-1 immunotherapy through T cells in CIN^+^ colorectal tumors.

## Discussion

KIF18A plays an important role in the tumorigenesis of various cancers. In breast cancer, KIF18A overexpression is correlated with tumor grade, metastasis and poor survival, while ablation of KIF18A dramatically restrains proliferation of breast cancer cells in vitro and in vivo via inhibiting PI3K/Akt pathway [23]. In glioblastoma, KIF18A inhibition suppresses cell growth, migration and invasion, and induces G2/M cell-cycle arrest of glioblastoma cells via interacting with PPP1CA [24]. KIF18A is upregulated in esophageal cancer patients, while silencing KIF18A decreases proliferation, migration and invasion of esophageal cancer cells [25]. In colorectal cancer, KIF18A is overexpressed and correlated with tumor stage, lymph node metastasis and peritoneal dissemination, while forced KIF18A expression promotes proliferation, migration and invasion of colorectal cancer cells [26, 27]. Correspondingly, we found that KIF18A inhibition apparently restrained proliferation, metastasis and tumor formation, and induced G2/M cell-cycle arrest of CIN^+^ colorectal cancer cells. Specifically, we proved that small inhibitors of KIF18A were feasible to suppress the malignant phenotypes of CIN^+^ colorectal cancer cells, which provided therapeutic evidences for using KIF18A as a novel target in the treatment of CIN^+^ colorectal cancer. Moreover, we found that KIF18A inhibition enhanced immune infiltration and activation in CIN^+^ colorectal tumors. Indeed, a variety of studies reveal that KIF18A expression is correlated with immune infiltration in cancers. For example, high KIF18A expression is correlated increased infiltration of cytotoxic T cells, dendritic cells, NK cells, T helper cells, Th2 cells, and macrophages in glioma samples [18]. In addition, a comprehensive pan-cancer analysis reveals that KIF18A is correlated immune infiltration and immune checkpoint genes in a majority of cancers [17]. In our study, we found that KIF18A inhibition by AM-1882 enhanced infiltrating of CD45^+^ leukocytes, CD3^+^ T cells, CD3^+^CD8^+^ Cytotoxic T (Cyto T) cells, and decreased infiltrating of CD25^+^FoxP3^+^ regulator T (Treg) cells in CIN^+^ colorectal tumors.

Emerging evidences indicate that CIN^+^ tumors are “immune cold”, exhibit an immune exclusive signature and are refractory to anti-PD-1 immunotherapy [9, 28]. For example, hepatocellular carcinomas with CIN features exhibit immune-excluded traits, including low immune infiltration and alternations in antigen-presenting machinery [29]. In chromosomally unstable metastatic cancer cells, ectonucleotidase ENPP1 reduces immune cell infiltration, and promotes metastasis and resistance to anti-PD-1/PD-L1 treatment via selectively degrading extracellular cGAMP [30]. In breast cancer, high-CIN tumors exhibit increasing Arg1^+^ macrophage infiltration, accumulation of NK cells with reduced effector function, and increased resting regulatory T cell infiltration [31]. In our study, KIF18A inhibition stimulated pro-inflammatory type I IFN signaling activation in CIN^+^ colorectal tumors, suggesting that KIF18A inhibition changed CIN^+^ tumors from “immune cold” to “immune hot”. Indeed, KIF18A inhibition enhanced infiltration and activation of cytotoxic T cells and decreased the infiltration and proliferation of Treg cells, thus augmented antitumor immunity in CIN^+^ colorectal tumors. This is corresponding with other studies. They demonstrate that targeting cell-cycle machinery may activate IFN signaling in tumors. For example, CDK4/6 inhibition stimulates type III interferons production in breast carcinoma and other solid tumors [16]. Dinaciclib, a potent CDK1, -2, -5 and -9 inhibitor, induces a type I IFN gene signature in various tumor cells [32]. YKL-5-124, a potent CDK7 inhibitor, activates IFN-γ signaling and induces TNF-a and CXCL9/10 in small-cell lung cancer [33]. In our study, KIF18A inhibition by AM-1882 promoted cGAS-STING pathway activation in CIN^+^ colorectal tumors. We speculated that KIF18A inhibition might induce double-strand DNA (dsDNA) production by disrupting cell-cycle progression, thus activated cGAS-STING pathway, which is a critical sensor for dsDNA in cells. Indeed, we found that KIF18A inhibition induced G2/M arrest of CIN^+^ colorectal tumors.

Accumulated studies indicate that CIN^+^ colorectal tumors are refractory to anti-PD-1 immunotherapy [28, 34]. However, KIF18A inhibition enhanced response to anti-PD-1 immunotherapy in CIN^+^ colorectal tumors in our study. Our data elucidated a novel role of KIF18A in antitumor immunity and anti-PD-1 immunotherapy in CIN^+^ colorectal cancer. Actually, targeting cell-cycle machinery may modulate antitumor response and PD-1 blockade efficiency in cancers. For instance, CDK7 inhibition by a small inhibitor YKL-5-124 induces DNA replication stress, genome instability and a robust immune surveillance program elicited by T cells, thus a combination of YKL-5-124 and anti-PD-1 shows evident survival benefit in small cell lung cancer [33]. Dinaciclib potentiates anti-PD1 immunotherapy via increasing T cell infiltration, DC activation and immunogenic cell death in immunocompetent mouse tumor models [32]. In ovarian cancer, CD4/6 inhibition by abemaciclib improves anti-PD-1 response via driving CD8^+^ cytotoxic T cell and B cell recruitment to the tumor microenvironment [35]. In our study, the combination of anti-PD-1 mAb and AM-1882 increased the infiltration of CD3^+^CD8^+^ Cyto T cells, and decreased the infiltration of Tregs in CIN^+^ colorectal tumors. Moreover, depletion of either CD4^+^ T cells or CD8^+^ T cells completely ablated the antitumor activity of the combine treatment of anti-PD-1 mAb and AM-1882. These results indicated that enhanced PD-1 blockade efficiency by KIF18A inhibition was depended on T cells.

In summary, we found that KIF18A inhibition suppressed growth and metastasis, and induced G2/M cell-cycle arrest of CIN^+^ colorectal cancer cells. Moreover, KIF18A inhibition promoted immune infiltration and activation in CIN^+^ colorectal cancer cells, thus augmented antitumor immunity. Mechanically, KIF18A inhibition stimulated type I IFN signaling and cGAS-STING activation. Targeting KIF18A enhanced response to anti-PD-1 immunotherapy through T cells in CIN^+^ colorectal tumors. Our data elucidated a novel role of KIF18A in antitumor immunity of CIN^+^ colorectal cancer.

## Materials and methods

### Cell culture and reagents

Human colorectal cancer cell lines NCI-H747, SW620 and HCT-166, and mouse colorectal cancer cell lines CT26 and MC38 were obtained from institute of Biochemistry and Cell Biology at the Chinese Academy of Sciences (Shanghai, China). Cells were cultured in Dulbecco’s Modified Eagle Medium (DMEM, Gibco, USA) supplemented with 10% fetal bovine serum (FBS, Gibco, USA) at 37 °C containing 5% CO_2_ in a humidified atmosphere. AM-1882 were purchased from Amgen (USA) and dissolved in DMSO for use unless otherwise indicated. VLS-1488 was purchased from Selleck Chemicals (Selleck #E4602, USA) and dissolved in DMSO for use unless otherwise indicated.

### Plasmid constructs

To deplete KIF18A expression, short hairpin RNAs (ShRNAs) targeting human or mouse KIF18A (Sh#1 and Sh#2) were synthesized and introduced into the pLKO.1 plasmid. The plasmid introduced with a non-targeting sequence was regarded as scramble control (SCR). The sequences for ShRNAs were: Sh#1: 5′-ACGCA TTCGA CGGGA TAATTC-3′ for mouse, and 5′-ATTCT ACGAT GACAC ATATAA-3′ for human; Sh#2: 5′-GTGGT TCATG TAGTG GATAAA-3′ for mouse, and 5′-CCCAT CTTAA AGCTA AGTTTA-3′ for human; SCR: 5′-ACGGA GGCTA AGCGT CGCAA-3′.

### Western blot

Cultured cells and tissue samples were lysed with RIPA lysis buffer (Beyotime, China) supplemented with protease inhibitors (Sigma, USA). Protein concentration was measured by BCA kit (Beyotime, China). Equivalent amounts of protein were separated by 8%-10% SDS-PAGE and transferred to PVDF membranes (Millipore, USA). The membranes were blocked by 5% non-fat milk, incubated with first antibodies at 4 °C overnight and corresponding second antibodies at room temperature for 1 h. Western blot bands were revealed by ECL kit (ThermoFisher Scientific, USA). The antibodies used in this study were list below: KIF18A (Bethyl Laboratories #A301-080A, USA), GAPDH (Cell signaling technology #2118, USA), p21 (Cell signaling technology #37543, USA), CDK1 (Abcam #ab265590, USA), Cyclin B1 (Cell signaling technology #4135, USA), STAT1 (Cell signaling technology #9172, USA), Phospho-Stat1 (Tyr701) (Cell signaling technology #9167, USA), TBK1 (Cell signaling technology #3013, USA), Phospho-TBK1 (Ser172) (Cell signaling technology #5483, USA), IRF3 (Cell signaling technology #4302, USA), Phospho-IRF-3 (Ser396) (Cell signaling technology #4947, USA). All antibodies were used as a dilution of 1: 1000.

### Cell viability assay

Viability of cells was determined by Cell Counting Kit-8 (Takara, Japan). Briefly, colorectal cancer cells were seeded in 96-well-plates (3000/well). Viability of cells was measured at day 2, 4 and 6. CCK-8 solution was added to each well (10 μL/well) and incubated at 37 °C for 2 h. Then, absorbance at 450 nm was determined by a microplate reader. All samples had three repeats.

### Transwell migration and invasion assays

Transwell migration and invasion assays were performed using transwell chamber (Millipore, USA). To evaluate cell migration, 3.0×10^4^ cells were seeded into the upper chamber in serum-free condition. The lower chamber was filled with 500 μL DMEM medium supplemented with 10% FBS. Cells were allowed to migrate for 24 h, then fixed by 4% paraformaldehyde for 15 min at room temperature and stained with crystal violet for 15 min. To evaluate cell invasion, the transwell chamber was pre-coated with Matrigel (BD, USA). Images of migration or invasion cells were taken by a microscope (Leica, Germany).

### Flow cytometry

Tissue samples were cut into small pieces and digested with dissociation buffer (2 mg/ml collagenase IV and 0.02 mg/ml DNase, Sigma, USA) in DMEM medium containing 10% FBS and PenStrep (Hyclone, USA) at 37 °C for 1 h with agitation. Cultured cells were digested with 0.05% trypsin for 5 min. Single cell suspensions were passed through 70 μm strainer. A total of 1.0 × 10^6^ cells were stained with appropriate antibodies diluted in PBS supplemented with 2% FBS at 4 °C for 30 min. For intracellular staining, cells were fixed and permeabilized using the Fixation/Permeabilization Solution Kit (BD bioscience, USA) as protocol described. To evaluate cell apoptosis, cells were stained with Annexin-V-FITC (Sigma, USA) and propidium iodide (PI) (Sigma, USA) for 10 min at room temperature in a dark room. To evaluate cell-cycle distribution, cells were stained with PI for 10 min avoiding light. Flow cytometry was performed using a B.D. Influx cell sorter (BD Bioscience, USA). Flow cytometry data were analyzed by Flowjo v10 software (Treestar, USA). The antibodies used in flow cytometry were: CD45 APC (ThermoFisher Scientific #MHCD4505), CD3 FITC (ThermoFisher Scientific #MHCD0301), CD4 PE (ThermoFisher Scientific #12-0041-82), CD8a V450 (BD biosciences #655111), CD25 PE-CY7 (ThermoFisher Scientific #25-0251-82), FoxP3 PerCP-CY5.5 (ThermoFisher Scientific #45-5773-82), CD69 APC (BD biosciences #340560), IFN-γ FITC (ThermoFisher Scientific #11-7319-82), CD86 FITC (ThermoFisher Scientific #MHCD8601), MHCII PerCP-Cyanine5.5 (ThermoFisher Scientific # A14902), CD11c APC (ThermoFisher Scientific # 17-0114-82), CD107a PE-CY7 (BD biosciences #560647), Ki67 (BD biosciences # 568147), Granzyme B (BD biosciences # 570499), PD-1 (BD biosciences #562671), Tim-3 (BD biosciences #747619).

### Animal models

Animal studies were reviewed and approved by the Institutional Animal Care and Use Committee of The Chinese PLA General Hospital (Approval number: HZKP-PJ-2023-26). For lung metastasis model, luciferase-labeled CT26 cells (1.0 × 10^5^) transduced with scramble (SCR) or KIF18A Sh#1 virus were injected into the tail vein of 6-week-old female ICR mice (n = 5 for each group), then tumors were allowed to grow for three weeks. For liver metastasis model, luciferase-labeled CT26 cells (1.0 × 10^5^) transduced with SCR or KIF18A Sh#1 virus were injected into the spleen of 6-week-old female ICR mice (*n* = 5 for each group), then tumors were allowed to grow for three weeks. Bioluminescence of tumor-bearing mice was measured by the IVIS Spectrum (PerkinElmer) at 5 min after intraperitoneal injection of 100 μL D-luciferin (10 mg/mL, Promega, USA). Images were analyzed by the Living Image 3.2 software package (Caliper Life Sciences). For tumor xenograft growth formation assay, CT26 cells (5.0 × 10^5^) were subcutaneously injected into 6-week-old female ICR mice (*n* = 5 for each group), then treated with 10 mg/kg and 25 mg/kg AM-1882 or 20 mg/kg VLS-1488 (dissolved in 2% hydroxypropyl methylcellulose and 1% Tween-80 at pH 2.2) by gavage every other day for three weeks. Mice in vehicle group (Veh) were treated with equal volume of 2% hydroxypropyl methylcellulose and 1% Tween-80 at the same time. To test the influence of αPD1, αCD4 and αCD8 antibodies on tumor xenograft growth of AM-1882 treated CT26 tumors, CT26 cells (5.0 × 10^5^) were subcutaneously injected into 6-week old female ICR mice (n = 6 for each group) for one week, then treated with 10 mg/kg AM-1882 (dissolved in 2% hydroxypropyl methylcellulose and 1% Tween-80 at pH 2.2) by gavage every other day, and 250 μg antibodies against PD1, CD4 and CD8 (BioXCell, USA) or IgG2b control isotype intraperitoneally every 3 days for 7 times as indicated. Tumor volume was measured by caliper every three days and calculated by the equation: volume = (length × width^2^)/2. At the end of drug treatment, mice were anaesthetized by 3% isoflurane and sacrificed by neck dissociation method. Tumor xenografts were dissected out and weighed.

### ADP-Glo motor assay

ADP-Glo motor assay was conducted as previously described using the ADP-Glo luminescence assay kit (Promega #v9101, USA) [11]. KIF18A recombinant protein was purchased from CUSABIO biotechnology (China). KIF18A small inhibitor AM-1882 (0–3000 nM) were assessed with 300 mM ATP, 30 μg/mL MTs and 100 μg/mL KIF18A recombinant protein. The luminescence was recorded by a plate reading luminometer. AM-1882 MT-ATPase IC_50_ values were calculated. Each sample had three repeats.

### Immunohistochemistry (IHC) staining

Tumor tissues were fixed by formalin at room temperature overnight, embedded by paraffin and cut into 8 μm sections. The sections were deparaffinized by xylene and rehydrated by ethanol gradients. Antigen retrieval was performed by heating with sodium citrate buffer. Sections were incubated with CD4 (Cell Signaling Technology #25229, 1:50), CD8 (Cell Signaling Technology #98941, 1:50), and FoxP3 (Cell Signaling Technology #12653, 1:50) at 4 °C overnight. Then, sections were treated with horseradish peroxidase-coupled secondary antibody at 4 °C for 1 h, and revealed by the ChemMate Envision detection system (Dako Cytomation).

### Transcriptome RNA-sequencing and GO analysis

Total RNA was extracted by TRIzol reagent (Takara, Japan) and purified by the Ribo-off Rrna Depletion Kit (Vazyme #N406, USA). VAHTS Universal V8 RNA-seq Library Prep Kit for lllumina (Vazyme #NR605, USA) was used to synthesize Cdna library, then sequenced on the Illumina HiSeq2500 platform (Illumina, USA). Gene expression was calculated by RPKM value. Differentiated expression genes were defined as |log_2_ Fold Change| ≥ 1.5 and adjusted *p* < 0.05. The significant affected signaling pathways were evaluated by gene ontology (GO) analysis using DAVID 6.8 tools. All samples were conducted with three repeats.

### Reverse transcription-quantitative PCR (RT-qPCR)

Total RNAs were extracted using the TRIzol reagent (Takara, Japan). The complementary DNA (cDNA) was synthesized by the high-capacity RNA to cDNA kit (ThermoFisher Scientific #4387406, USA). Quantitative PCR was performed using the PowerUp SYBR Green Master Mix (ThermoFisher Scientific #A25742, USA) on the ABI 7900HT quantitative PCR system (ABI biosystems, USA). Gene expression was normalized to GAPDH and calculated by 2^−ΔΔCq^ method. The primers used in this study were listed in Supplementary Table 1. All samples had three repeats.

### ELISA assay

The levels of TNF-α and IFN-γ in CT26 tumors were determined by ELISA kits (Abcam #ab208348 and Abcam #ab252363, USA) as protocol described. Briefly, samples were incubated with antibody-coated plates, biotinylated antibodies, streptavidin antibodies, substrate solution and stop solution consecutively as protocol indicated. Then, optical density at 450 nm was measured by a microplate reader. All samples had three repeats.

### Statistical analysis

Statistical analysis was performed using the GraphPad Prism 8.0 software (GraphPad Software, CA). Difference between groups was evaluated by two tailed Student’s *t* test and One-way ANOVA (Bonferroni’s post-hoc test). Data were shown as mean ± SD and *p* < 0.05 was regarded as statistically significant.

## Supplementary information

Supplementary figure legends

Supplementary Figure 1

Supplementary Figure 2

Supplementary Figure 3

Supplementary Figure 4

Supplementary Figure 5

Supplementary Figure 6

Supplementary Figure 7

Supplementary table 1

Original western blot gels

## Data Availability

The data that support the findings of this study are available on request from the corresponding author.
