# Supplementary material for: Targeting KIF18A triggers antitumor immunity and enhances efficiency of PD-1 blockade in colorectal cancer with chromosomal instability phenotype
Source: Cell Death Discov. 2025 Apr 2;11:130. doi: 10.1038/s41420-025-02437-5 (PMC11965295; doi:10.1038/s41420-025-02437-5)
Supplement: Supplementary file 1 — Supplementary figure legends [file 41420_2025_2437_MOESM1_ESM.docx]

**Supplementary figure legends**

**Supplementary Figure 1. Silencing KIF18A showed no apparent influence on growth, migration, invasion and apoptosis of CIN^-^ colorectal cancer cells.** A, HCT-116 and MC38 cells introducing with SCR, Sh#1 and Sh#2 lentivirus were seeded into 96-well-plates (3000/well), then cell viability was determined by Cell Counting Kit-8 at day 2, 4 and 6. B-C, HCT-116 and MC38 cells introducing with SCR, Sh#1 and Sh#2 lentivirus were used for transwell migration and invasion assays. Representative images (B) and relative migration and invasion cells (C) were shown. D-E, MC38 cells introducing with SCR, Sh#1 and Sh#2 lentivirus were stained with PI and Annexin V-FITC for flow cytometry. Representative plots (E) and percentage of apoptotic cells (F) were shown. **P*＜0.05.

**Supplementary Figure 2. KIF18A inhibition showed no evident influence on cell cycle of CIN^-^ colorectal cancer cells.** A, HCT-116 and MC38 cells were introduced with SCR, Sh#1 and Sh#2 lentivirus, then collected lysates for western blot. B, HCT-116 and MC38 cells introducing with SCR, Sh#1 and Sh#2 lentivirus were stained with PI for flow cytometry. The percentages of cells in G0/G1, S and G2/M phases were shown. **P*＜0.05.

**Supplementary Figure 3. Gating strategy for flow cytometry analysis.**

**Supplementary Figure 4. The influence of KIF18A inhibition on T cells of mice bearing with/without CT26 tumors.** A, the percentages of infiltrating CD45^+^ leukocytes, CD3^+^ T cells, CD3^+^CD4^+^ Th cells, CD3^+^CD8^+^ Cyto T cells, and CD25^+^FoxP3^+^ Treg cells in CT26 tumors introducing with SCR, Sh#1 and Sh#2 lentivirus were shown. B, the percentages of CD3^+^ T cells, CD3^+^CD4^+^ Th cells and CD3^+^CD8^+^ Cyto T cells in spleen, liver, lung and colon tissues of mice bearing with/without CT26 tumors were evaluated by flow cytometry and shown. C, the percentages of CD69^+^CD4^+^ T cells and CD69^+^CD8^+^ T cells in spleen, liver, lung and colon tissues of mice bearing with/without CT26 tumors were evaluated by flow cytometry and shown. **P*＜0.05, n.s., not significant.

**Supplementary Figure 5. KIF18A inhibition exhibits no influence on immune infiltration of CIN^-^ colorectal tumors.** A, the percentages of infiltrating CD45^+^ leukocytes, CD3^+^ T cells, CD3^+^CD8^+^ Cyto T cells, and CD25^+^FoxP3^+^ Treg cells in MC38 tumors treating with 25 mg/kg AM-1882 or vehicle control (Veh) were shown. B-C, the percentages of CD69 (B), and IFN-γ (C) positive cells in infiltrating CD4^+^ and CD8^+^ T cell subsets of MC38 tumors treating with 25 mg/kg AM-1882 or vehicle control (Veh) were shown. n.s., not significant.

**Supplementary Figure 6. The influence of AM-1882 and VLS-1488 on immune cells *in vitro* and *in vivo*.** A-B, CD8^+^ T cells treating with 50 nM AM-1882 or vehicle control (Veh) were stained with Ki67 or Granzyme B (GranB) for flow cytometry. Representative histogram (A) and percentages of positive cells (B) were shown. C-D, bone marrow-derived CD11c^+^ dendritic cells were treated with 50 nM AM-1882 or vehicle control (Veh) for 72 h, then the percentages of CD86^+^ or MCHII^+^ cells were evaluated by flow cytometry. Representative histogram (C) and percentages of positive cells (D) were shown. E-F, infiltrating CD8^+^ T cell subsets in CT26 tumors treating with 20 mg/kg VLS-1488 or vehicle control (Veh) were stained with Ki67 or Granzyme B (GranB) for flow cytometry. Representative histogram (E) and percentages of positive cells (F) were shown. G-H, infiltrating CD8^+^ T cell subsets in CT26 tumors treating with 20 mg/kg VLS-1488 or vehicle control (Veh) were stained with PD-1 and TIM-3 for flow cytometry. Representative plots (G) and percentages of PD-1^+^ TIM3^+^ cells (H) were shown. I-J, infiltrating CD3^+^CD4^+^ T cell subsets in CT26 tumors treating with 20 mg/kg VLS-1488 or vehicle control (Veh) were stained with CD25 and FoxP3 for flow cytometry. Representative plots (I) and percentages of CD25^+^ FoxP3^+^ cells (J) were shown. **P*＜0.05, n.s., not significant.

**Supplementary Figure 7. Knockdown of KIF18A promotes IFN-γ and cGAS-STING signaling in CIN^+^ colorectal tumors. A-B,** relative expression of IFN-related transcription factors (A) and IFN-stimulated genes (B) in CT26 tumors introducing with SCR, Sh#1 and Sh#2 lentivirus were evaluated by RT-qPCR. C, the production of TNF-α and IFN-γ in CT26 tumors introducing with SCR, Sh#1 and Sh#2 lentivirus was evaluated by ELISA assay. D, the levels of phosphorylation and total TBK1 and IRF3 in CT26 tumors introducing with SCR, Sh#1 and Sh#2 lentivirus was evaluated by western blot. **P*＜0.05.
