# Supplementary figures and images for: Targeting KIF18A triggers antitumor immunity and enhances efficiency of PD-1 blockade in colorectal cancer with chromosomal instability phenotype
Source: Cell Death Discov. 2025 Apr 2;11:130. doi: 10.1038/s41420-025-02437-5 (PMC11965295; doi:10.1038/s41420-025-02437-5)

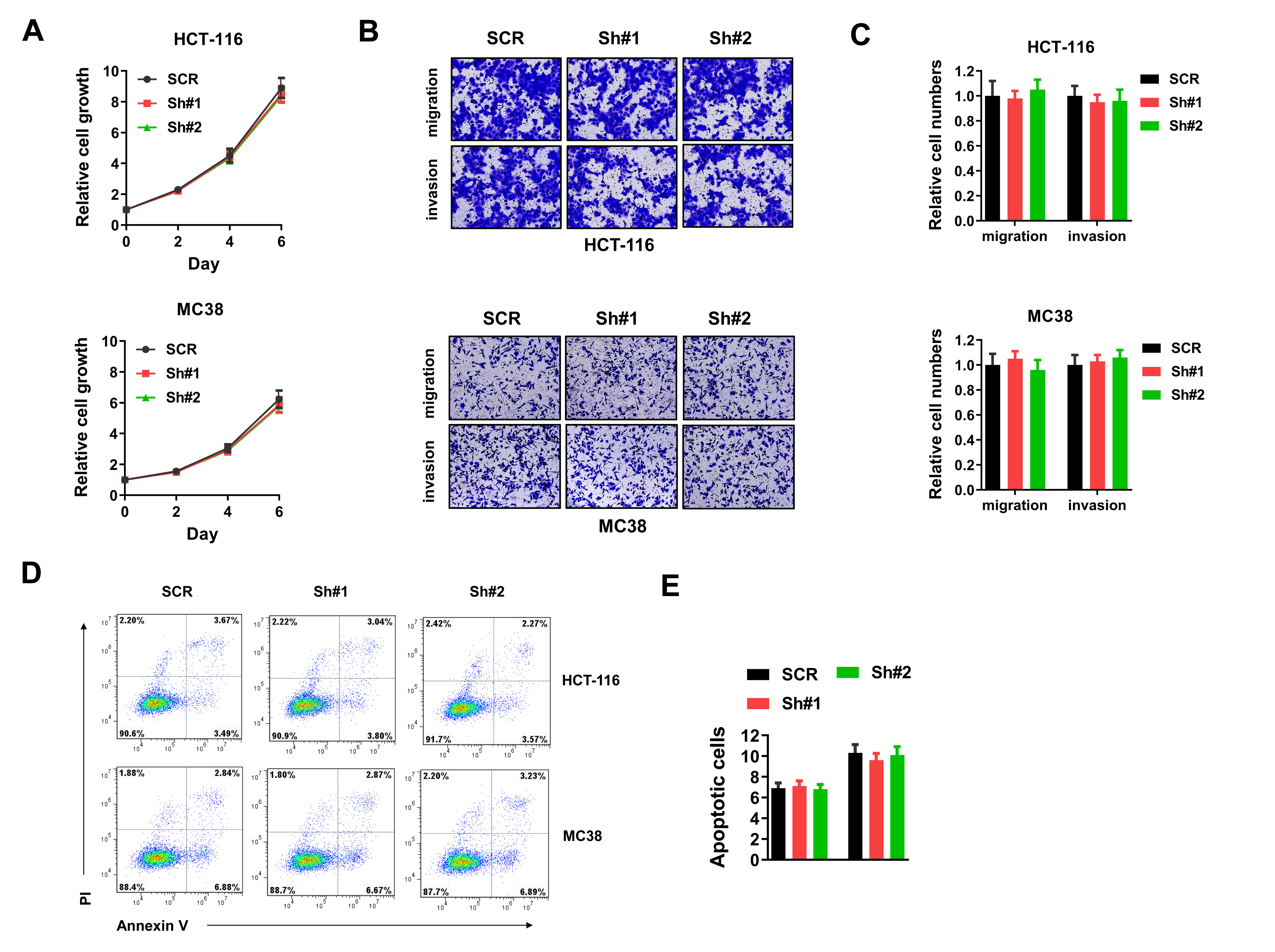

Supplement: Supplementary file 2 — Supplementary Figure 1 [file 41420_2025_2437_MOESM2_ESM.jpg]

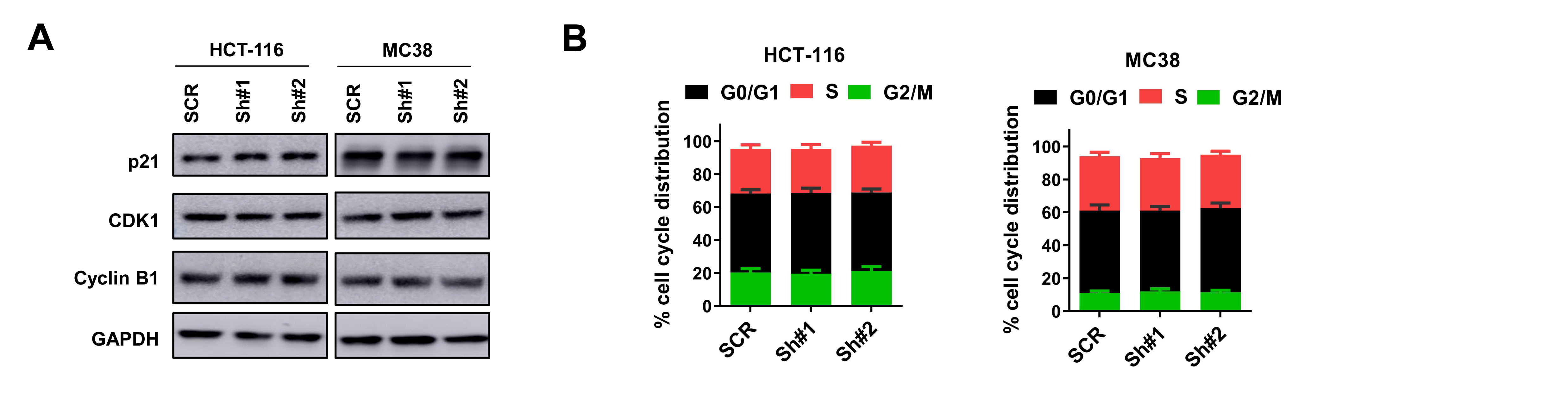

Supplement: Supplementary file 3 — Supplementary Figure 2 [file 41420_2025_2437_MOESM3_ESM.jpg]

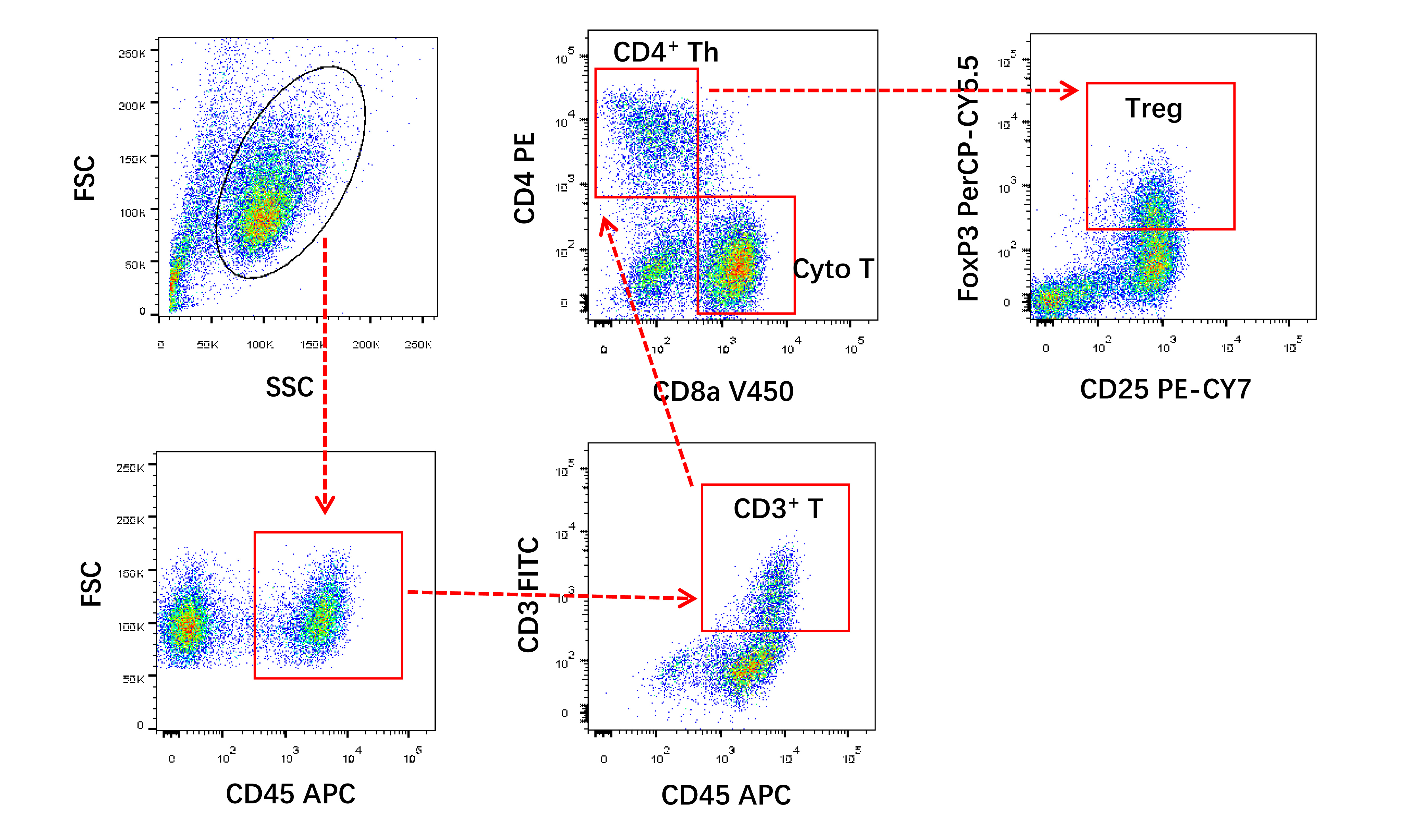

Supplement: Supplementary file 4 — Supplementary Figure 3 [file 41420_2025_2437_MOESM4_ESM.jpg]

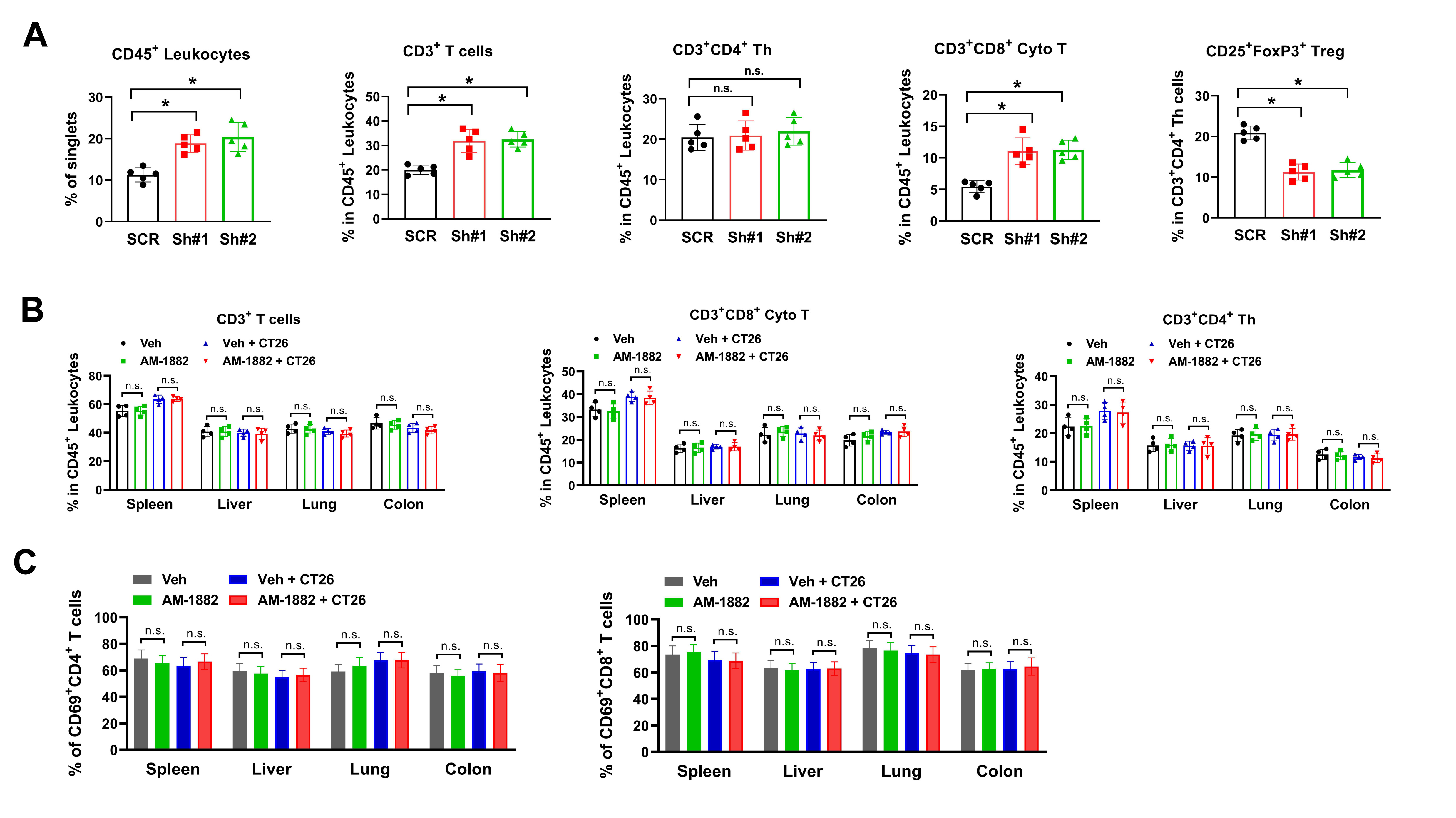

Supplement: Supplementary file 5 — Supplementary Figure 4 [file 41420_2025_2437_MOESM5_ESM.jpg]

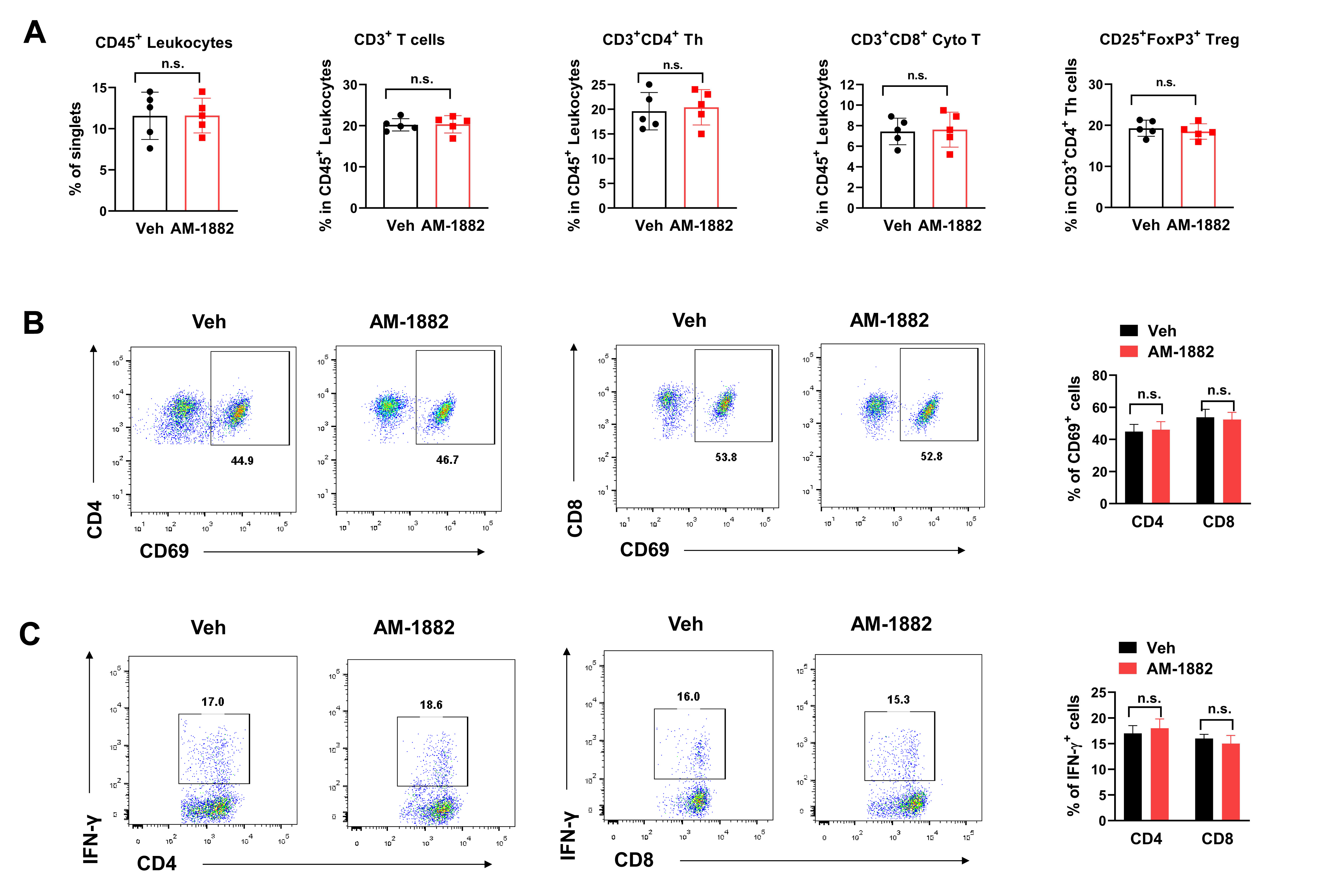

Supplement: Supplementary file 6 — Supplementary Figure 5 [file 41420_2025_2437_MOESM6_ESM.jpg]

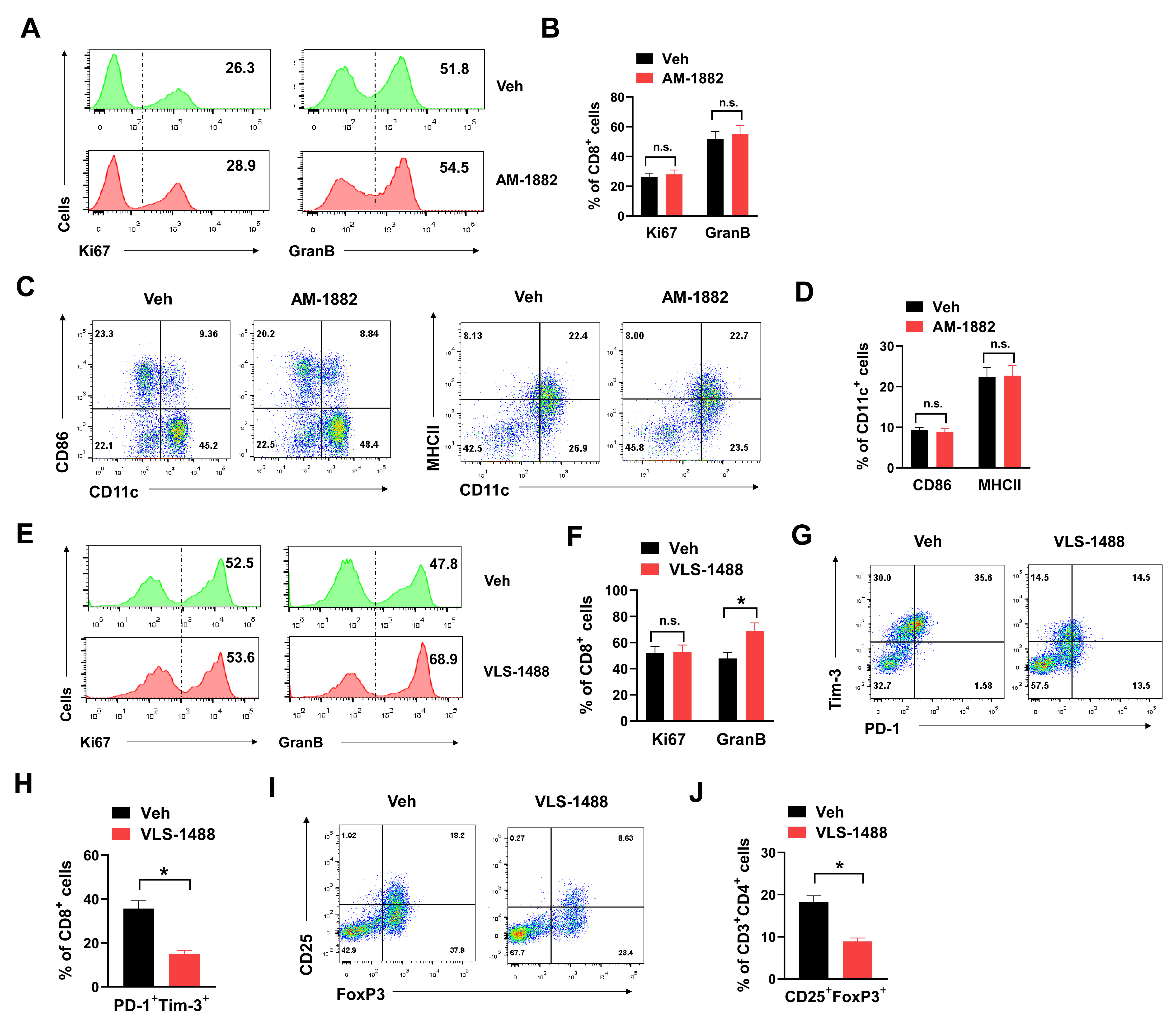

Supplement: Supplementary file 7 — Supplementary Figure 6 [file 41420_2025_2437_MOESM7_ESM.jpg]

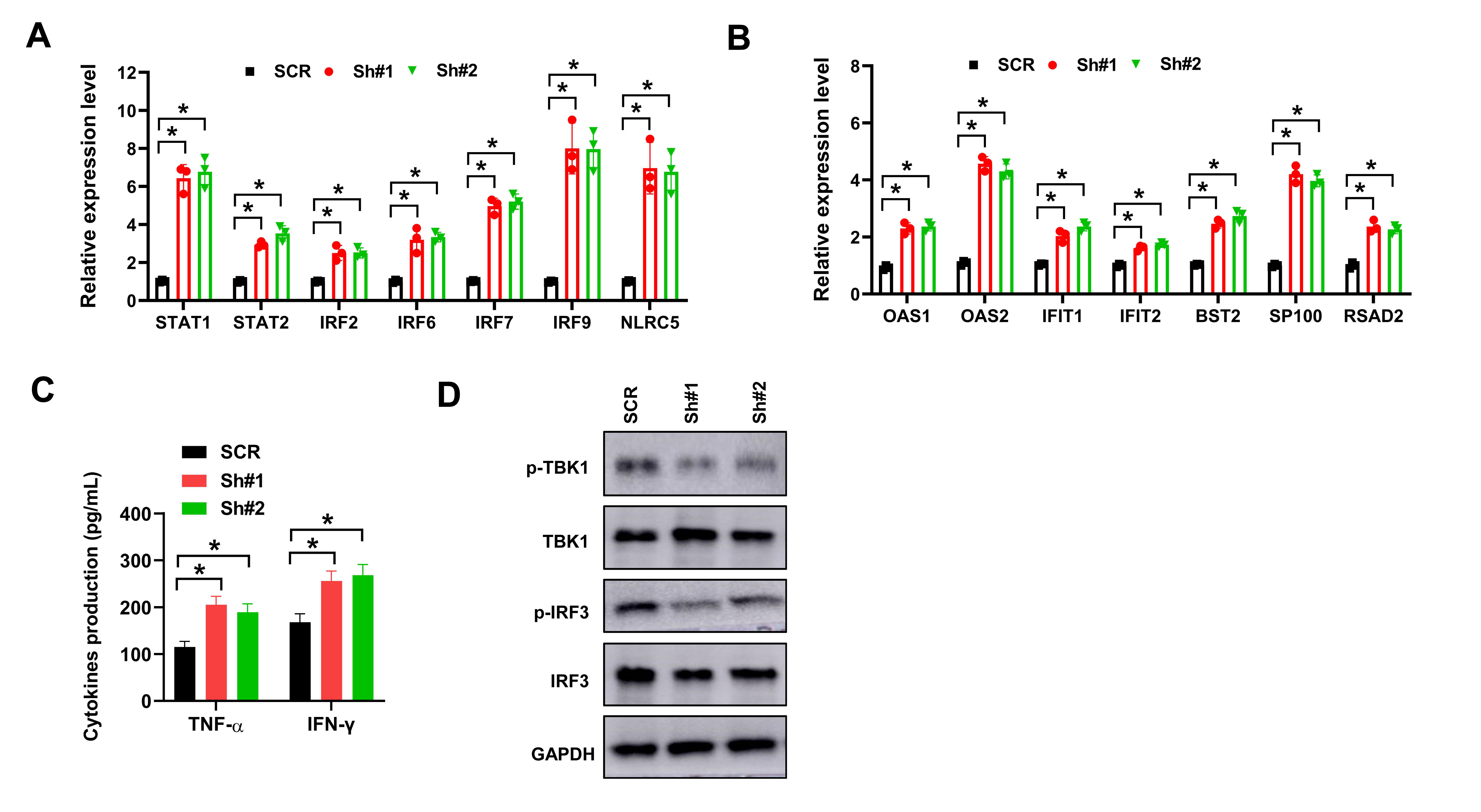

Supplement: Supplementary file 8 — Supplementary Figure 7 [file 41420_2025_2437_MOESM8_ESM.jpg]

### Figure 1A

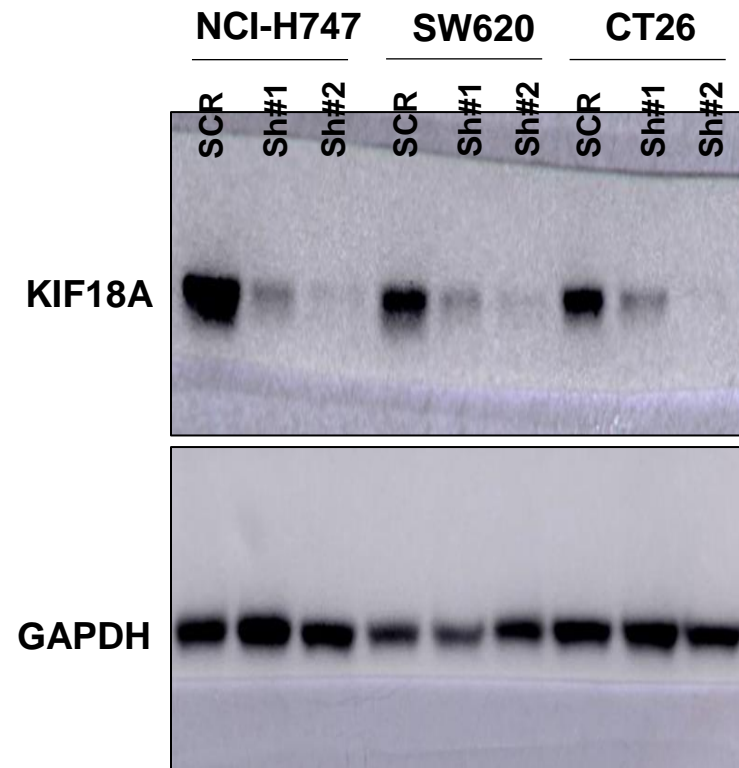

Figure 3A

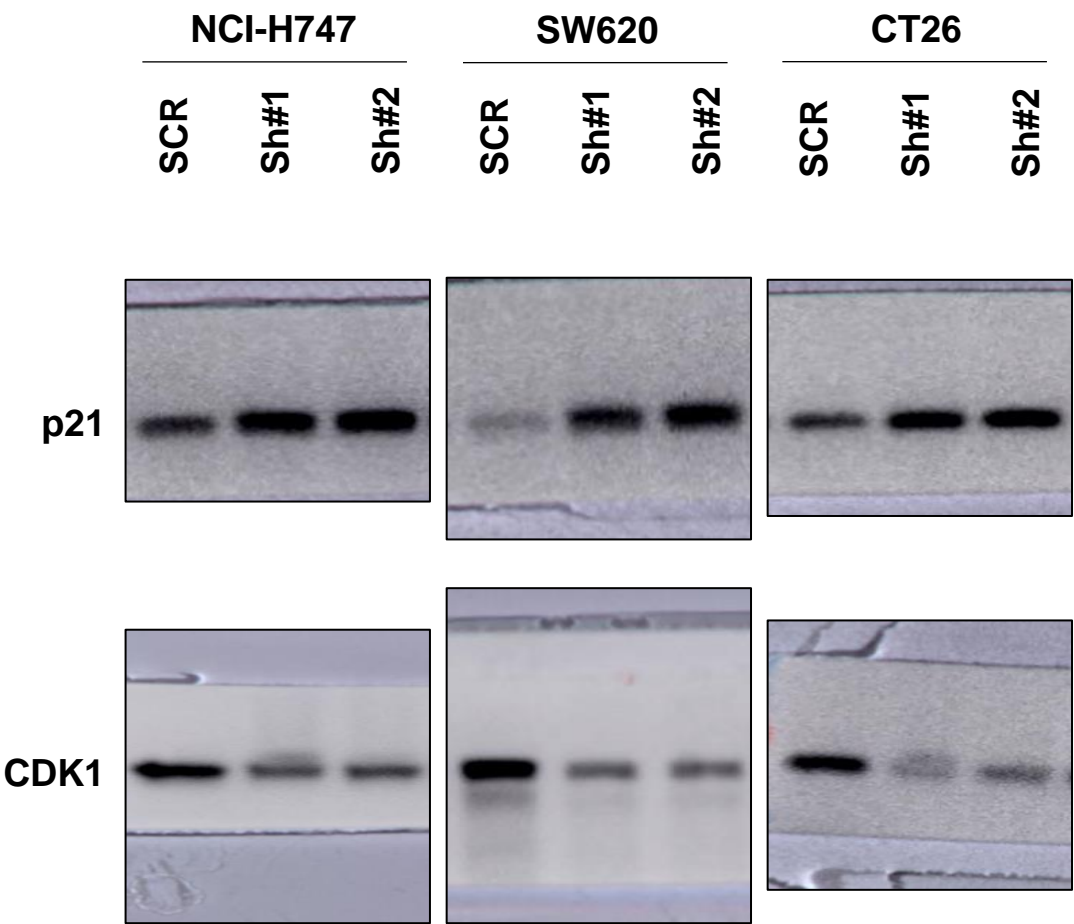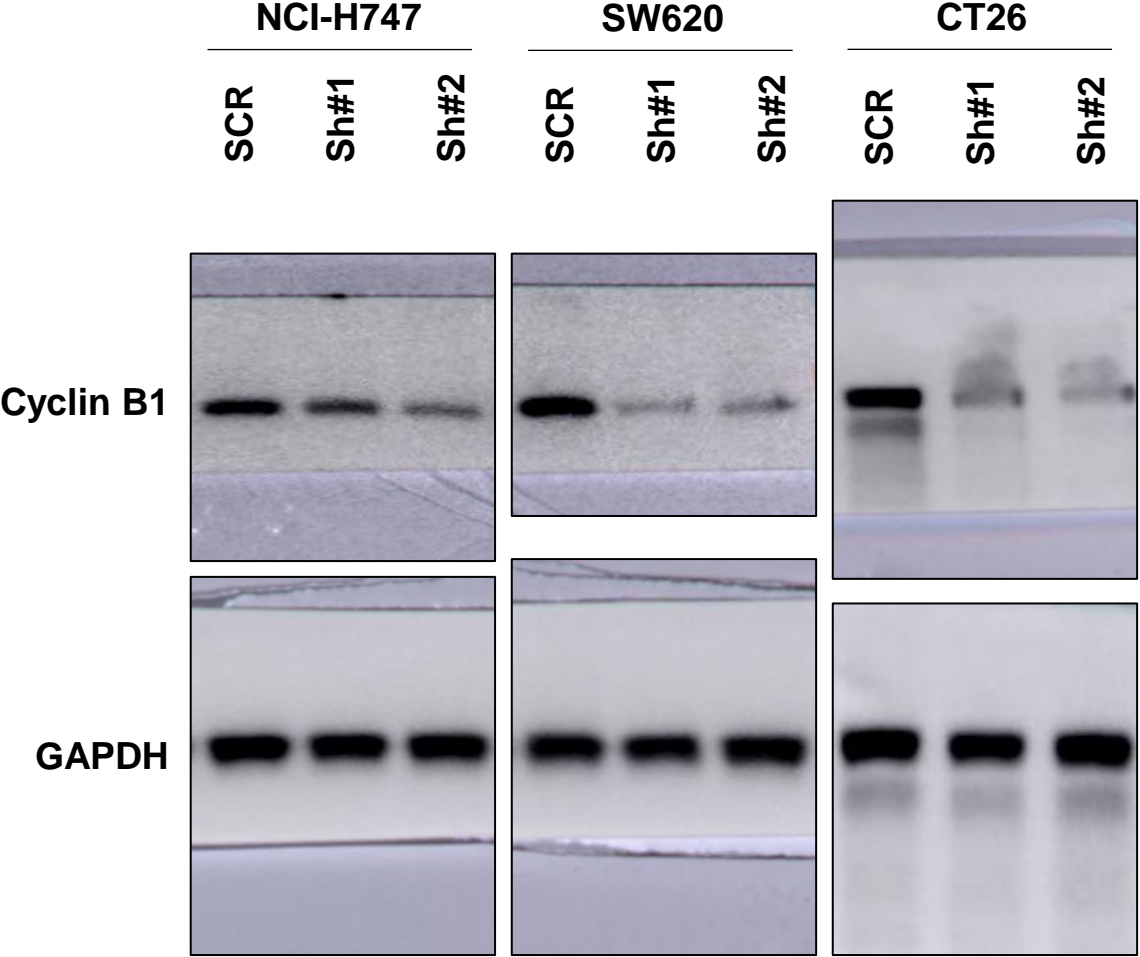

Figure 3C

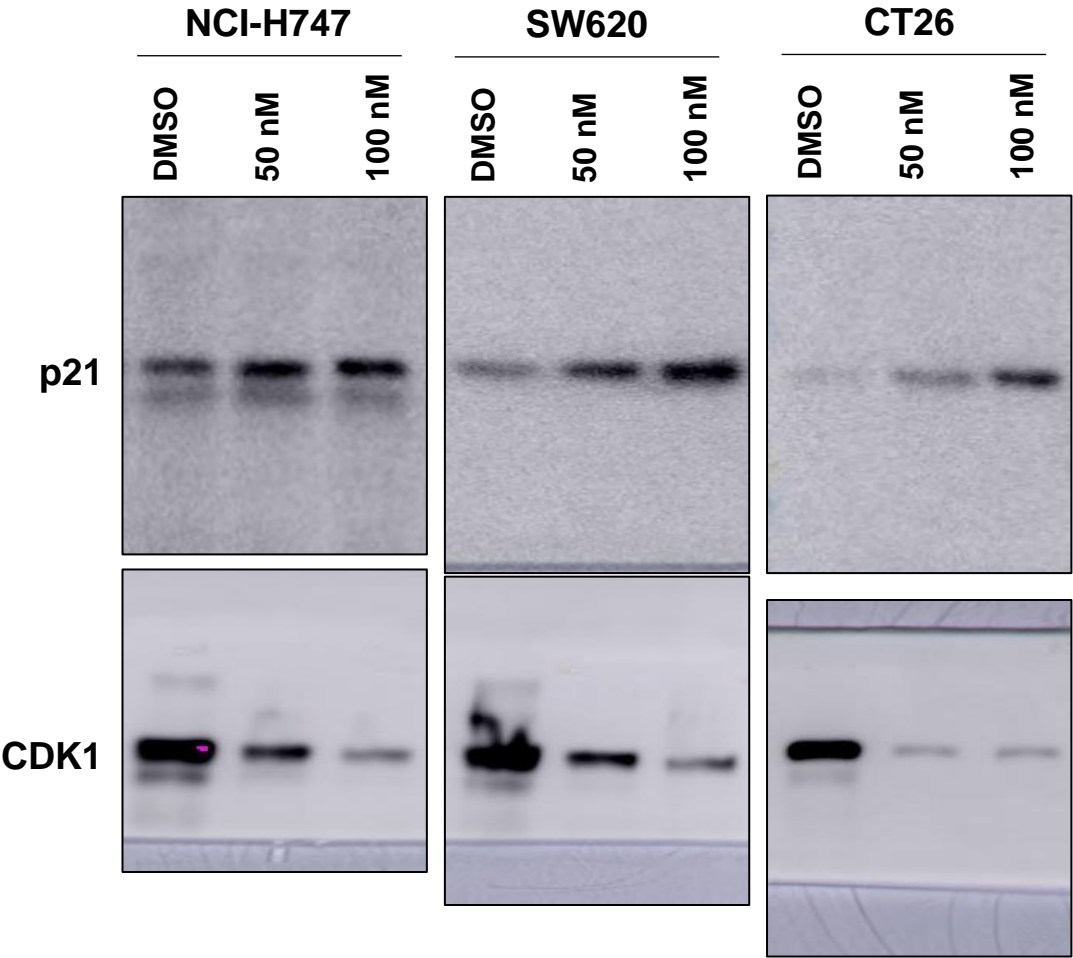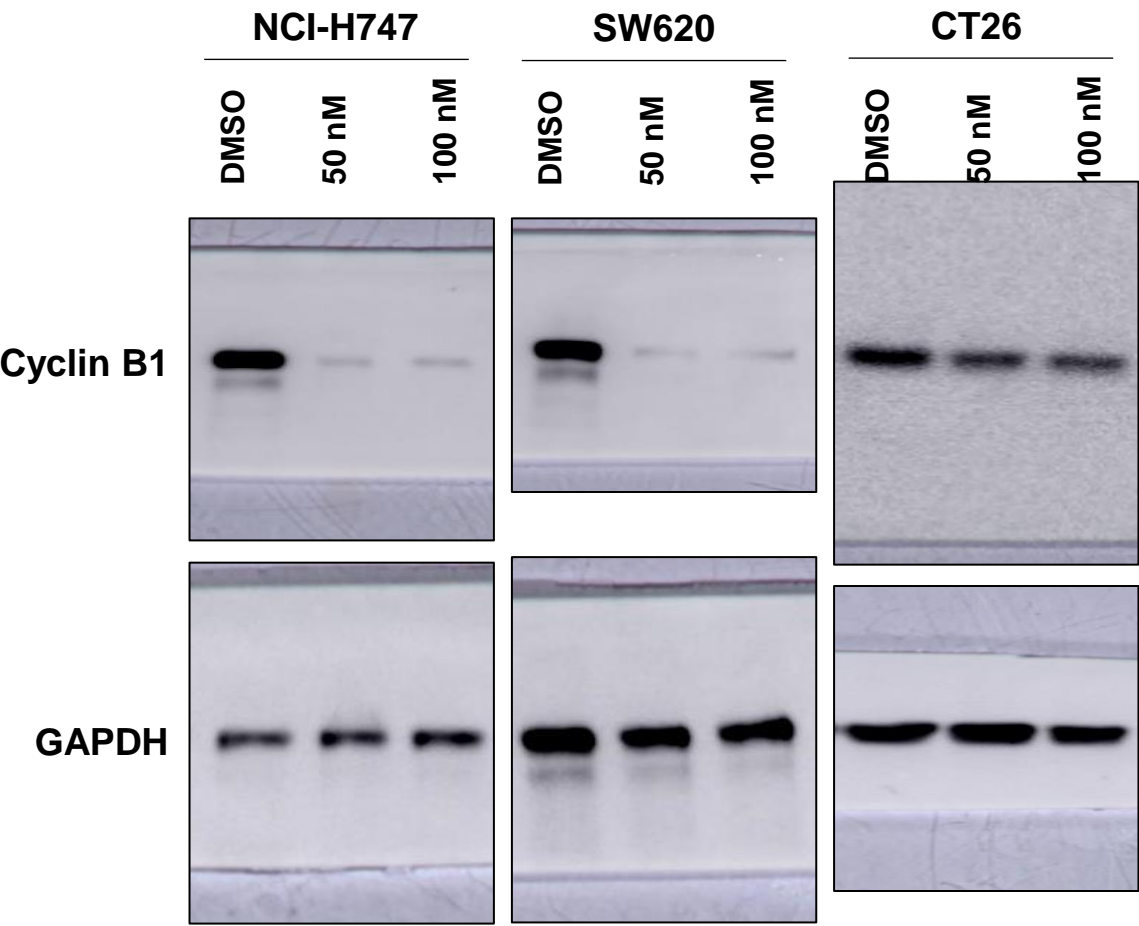

Figure 6E

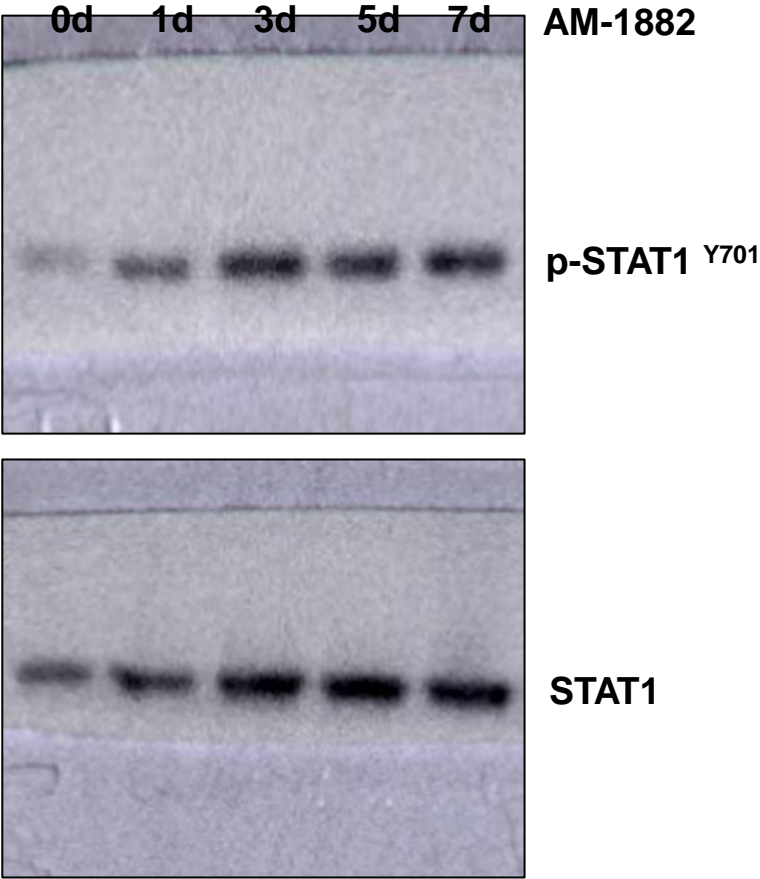

Figure 6G

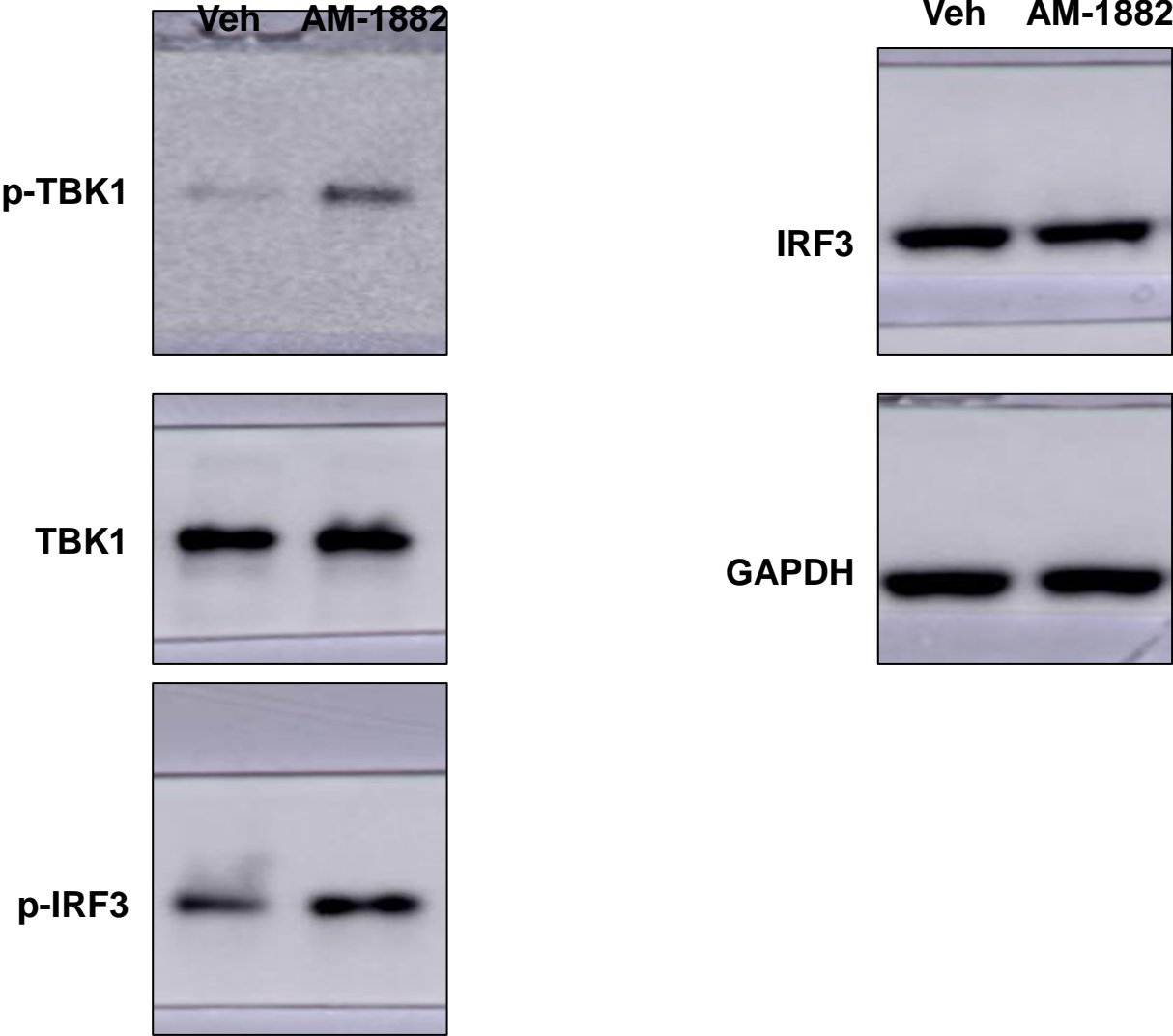

Figure S2

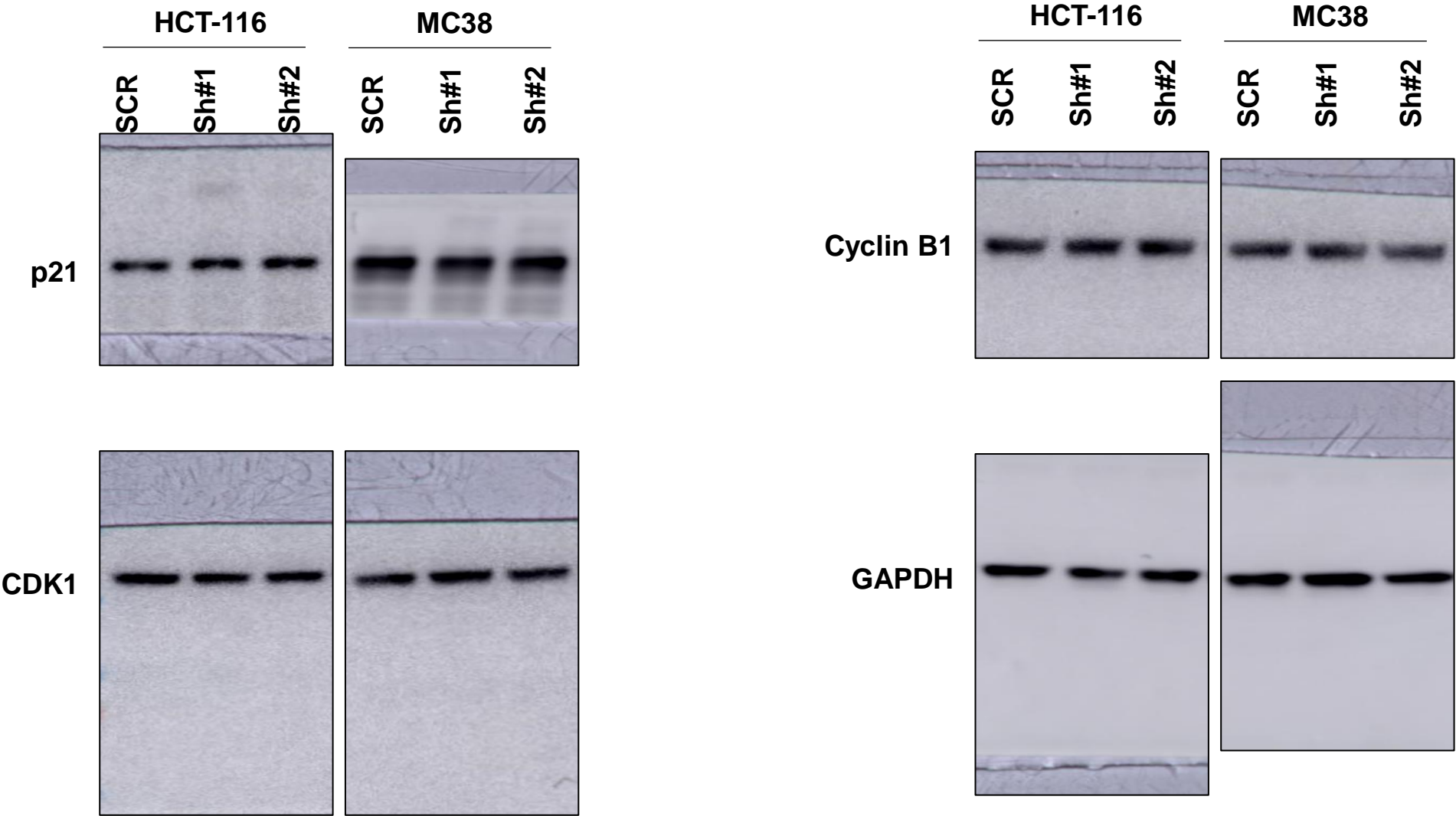

**Figure S7**

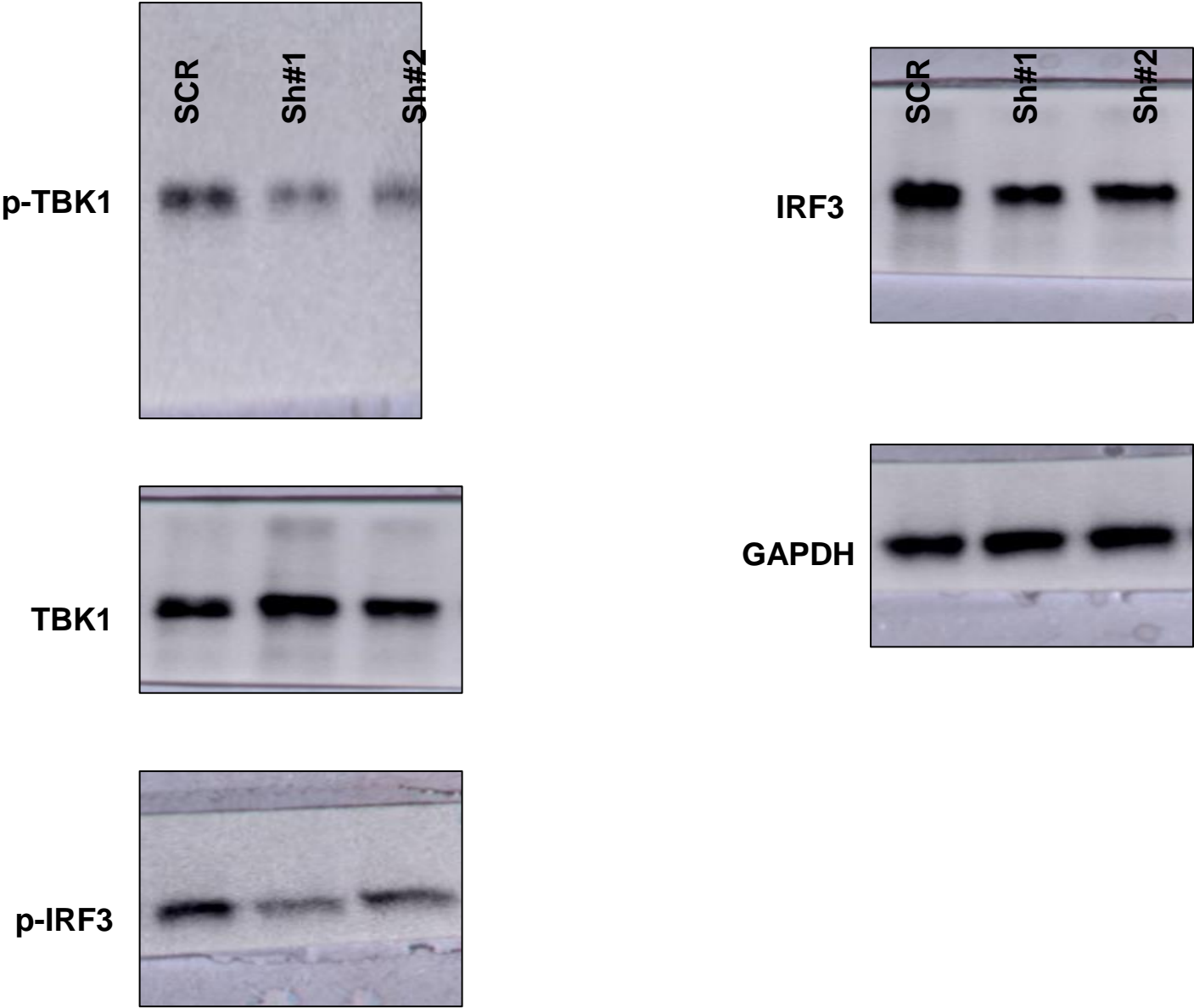

Supplement: Supplementary file 10 — Original western blot gels [file 41420_2025_2437_MOESM10_ESM.pdf]
