# Supplementary material for: Targeting KIF18A triggers antitumor immunity and enhances efficiency of PD-1 blockade in colorectal cancer with chromosomal instability phenotype
Source: Cell Death Discov. 2025 Apr 2;11:130. doi: 10.1038/s41420-025-02437-5 (PMC11965295; doi:10.1038/s41420-025-02437-5)
Supplement: Supplementary file 9 — Supplementary table 1 [file 41420_2025_2437_MOESM9_ESM.docx]

Supplementary Table 1. Primer sequences in RT-qPCR.

| Gene name | Forward primer | Reverse primer |
| --- | --- | --- |
| STAT1 | CACGCTGCCTATGATGTC | CTGGAGATTACGCTTGCTT |
| STAT2 | CAGTGACAGAGGAGTTACAT | GGCAATGGAGAGTTGGTT |
| IRF2 | AACAACGCCTTCAGAGTC | GTTCTTGCTTGATGTGCTTA |
| IRF6 | TGCTGCTCCTAACCTGAT | CGGCTGCTTCTCTATCTG |
| IRF7 | CTATCTGTGGCTACAACCTAA | AGATGCTACTACTCTGTGATG |
| IRF9 | TGCTTCTGTGATGGTTCTTA | CAAGTATCCTCAACAAGTCTG |
| NLRC5 | AGACTCTGCTTGACACATC | TACAGAAGGAGGTCAGGAG |
| OAS1 | CTGCTGAAGGAGGTGAAG | TGATGAGATTGGCGTAGAAT |
| OAS2 | GCTCGCACCTCTATTCTT | GGCAAGTGTCTCTGAAGG |
| IFIT1 | TTACTATGAGAAGGCACTGAG | GAAGGAGCATTGGAACACT |
| IFIT2 | GTCCTCTTGGCACTGAAG | CTCTGTCTGTGTCATATACCT |
| BST2 | GCTGGAGAATCTGAGGATC | AGAGCAGGAACAGTGACA |
| SP100 | GAAGGCTGGAGAAGTAATGA | GAGTTCACAGGAATCAAGGT |
| RSAD2 | TGGCTATCTACGGACTGTT | CTGTGGAGGACGATTATCTG |
| GAPDH | TCTCCTGCGACTTCAACA | TGTAGCCGTATTCATTGTCA |
